# *Mycoplasma gallisepticum* Lipid Associated Membrane Proteins Up-regulate Inflammatory Genes in Chicken Tracheal Epithelial Cells via TLR-2 Ligation through an NF-κB Dependent Pathway

**DOI:** 10.1371/journal.pone.0112796

**Published:** 2014-11-17

**Authors:** Sanjukta Majumder, Frank Zappulla, Lawrence K. Silbart

**Affiliations:** 1 Department of Animal Science, The University of Connecticut, Storrs, Connecticut, United States of America; 2 Department of Pathobiology and Veterinary Sciences, The University of Connecticut, Storrs, Connecticut, United States of America; 3 Center of Excellence for Vaccine Research, The University of Connecticut, Storrs, Connecticut, United States of America; 4 Department of Allied Health Sciences, The University of Connecticut, Storrs, Connecticut, United States of America; Miami University, United States of America

## Abstract

*Mycoplasma gallisepticum*-mediated respiratory inflammation in chickens is associated with accumulation of leukocytes in the tracheal submucosa. However the molecular mechanisms underpinning these changes have not been well described. We hypothesized that the initial inflammatory events are initiated upon ligation of mycoplasma lipid associated membrane proteins (LAMP) to TLRs expressed on chicken tracheal epithelial cells (TEC). To test this hypothesis, live bacteria or LAMPs isolated from a virulent (R_low_) or a non-virulent (R_high_) strain were incubated with primary TECs or chicken tracheae *ex vivo*. Microarray analysis identified up-regulation of several inflammatory and chemokine genes in TECs as early as 1.5 hours post-exposure. Kinetic analysis using RT-qPCR identified the peak of expression for most genes to be at either 1.5 or 6 hours. *Ex-vivo* exposure also showed up-regulation of inflammatory genes in epithelial cells by 1.5 hours. Among the commonly up-regulated genes were IL-1β, IL-6, IL-8, IL-12p40, CCL-20, and NOS-2, all of which are important immune-modulators and/or chemo-attractants of leukocytes. While these inflammatory genes were up-regulated in all four treatment groups, R_low_ exposed epithelial cells both *in vitro* and *ex vivo* showed the most dramatic up-regulation, inducing over 100 unique genes by 5-fold or more in TECs. Upon addition of a TLR-2 inhibitor, LAMP-mediated gene expression of IL-1β and CCL-20 was reduced by almost 5-fold while expression of IL-12p40, IL-6, IL-8 and NOS-2 mRNA was reduced by about 2–3 fold. Conversely, an NF-κB inhibitor abrogated the response entirely for all six genes. miRNA-146a, a negative regulator of TLR-2 signaling, was up-regulated in TECs in response to either R_low_ or R_high_ exposure. Taken together we conclude that LAMPs isolated from both R_high_ and R_low_ induced rapid, TLR-2 dependent but transient up-regulation of inflammatory genes in primary TECs through an NF-κB dependent pathway.

## Introduction

*Mycoplasma gallisepticum* (*M. gallisepticum*) is an avian respiratory pathogen causing severe inflammation of the trachea, air sacs and lungs, especially in the presence of a co-infection [1]–[3]. This pathogen is known to invade, survive and multiply inside a variety of non-phagocytic cells such as chicken RBCs, HeLa cells, and chicken fibroblasts, [4]–[9]. In addition, *M. gallisepticum* is known to colonize many extra-pulmonary tissues including blood, heart, spleen, liver and brain [4], [5], [7], [8], [10]. Indikova et al. (2013) suggested that invasion may occur at the air sac, where the mucosal barrier is quite thin [7]. However, there is yet no clear evidence that *M. gallisepticum* invades tracheal epithelial cells *in vivo* [unpublished observations], as it predominantly colonizes the mucosal surface and only rarely is found inside phagocytic vacuoles [11]. Nonetheless, the organism orchestrates immuno-pathological changes in the tracheal mucosa marked by infiltration of heterophils, macrophages and lymphocytes [2], [12], [13] soon after attachment and colonization of the respiratory surface.

A previous study from our laboratory reported up-regulation of several chemokines including lymphotactin, CXCL-13, RANTES and MIP-1β in chicken trachea isolated from live birds within 24 hours of experimental *M. gallisepticum* infection [12]. These chemokines are primarily produced by macrophages, lymphocytes and NK cells; cell types not found in large numbers in the uninfected tracheal mucosa [14]–[19]. However, chemokines and cytokines that are produced by epithelial cells upon infection are known for their ability to recruit phagocytic cells and lymphocytes into infected tissues [20]. Due to the protective layer of mucus, it is not clear if the initial interaction of mycoplasmas with the host epithelium is driven by viable organisms or microbial components such as lipoprotein-bearing membrane fragments or both, although substantial evidence supports the notion that the initial “pathogen perception” occurs upon interaction of various PAMPs with TLRs [20]–[24]. Previous studies conducted using other mycoplasma species suggest an important role for epithelial cells in inflammation. For example, A549 human lung epithelial cells increase their production of IL-8, TNF-α, IL-1β, and IL-6 following *Mycoplasma pneumoniae (M. pneumoniae)* exposure [25]. Similarly, cultured human endocervical epithelial cells exposed to *Mycoplasma genitalium (M. genitalium)* secreted several pro-inflammatory chemokines and cytokines including IL-6, IL-7, IL-8, MCP-1 and GM-CSF [26]–[28].

Due to the lack of a peptidoglycan cell wall or outer membrane, mycoplasmas do not possess lipopolysaccharides (LPS), lipotechoic acid or flagella. Even though certain mycoplasmas are known for production of exotoxins, like the *M. pneumoniae* CARDS toxin or *Mycoplasma arthritidis* mitogen MAM [29]–[32], the majority of mycoplasmas including *M. gallisepticum* are not known to produce or secrete any exotoxin. Their surface-exposed membranes are composed of a single lipid bi-layer with numerous embedded integral and peripheral proteins and membrane anchored lipoproteins [33]–[35]. Phase and antigenic variable expression of these membrane lipoproteins provides a mechanism of immune evasion [36]–[46], and the importance of these molecules is reflected by the percentage of the mycoplasma genome devoted to lipoproteins. For example, in *M. gallisepticum* about 10% of the genome is devoted to *vlhA*s (variable lipoprotein hemagglutinins) which includes 38 genes with signature *vlhA* features and 5 pseudogenes possessing *vlhA* sequence homology [47].

Mycoplasma lipoproteins are known to partition into the Triton X-114 detergent phase during phase partitioning. This detergent phase fraction may also contain other hydrophobic proteins besides lipoproteins [48], and therefore has been termed “lipid associated membrane proteins” (LAMPs) [48]–[51]. In other mycoplasma species, the detergent phase fraction containing these LAMPs was found to activate NF-κB via TLR-1, 2, 6 as well as CD-14 via a MyD88 pathway, and induce expression of pro-inflammatory cytokines in monocytes and macrophages [43], [48], [50]–[53]. Recently, it was also found that mycoplasma LAMPs are capable of activating the NLRP3 inflammasome resulting in the induction of IL-1β [54]. Several other studies found that lipoproteins purified from the TX-114 fraction induce inflammatory responses via TLR-2 or TLR-1/2 andTLR-2/6 heterodimers [28], [34], [48]–[50], [55]–[59]. However, the vast majority of these *in-vitro* studies were performed using leukocytes even though the initial interactions between mycoplasma membrane components and host cells occur at the mucosal surface upon contact with epithelial cells [28], [60], [61]. Thus, we hypothesized that LAMPs of *M. gallisepticum* ligate TLR-2 on respiratory epithelial cells, resulting in the up-regulation of inflammatory chemokine and cytokine genes via an NF- κB dependent pathway. To test this hypothesis, *M. gallisepticum* LAMPs were incubated with primary chicken tracheal epithelial cells in culture (TEC) or tracheae *ex vivo* to examine differential gene expression and to determine if the response is mediated via TLR-2 ligation. Similar studies using viable organisms were conducted to assess the relative contribution of LAMPs to early inflammatory events, using the low passage, adherent and virulent *M. gallisepticum* strain R_low_ or a high passage, non-adherent and non-virulent strain, R_high._

## Materials and Methods

### Bacterial Strains and Culture Conditions

*M. gallisepticum* low passage virulent strain R_low_ (passage 17) and high passage non-virulent strain R_high_ (passage 167) were cultured at 37°C in modified Hayflicks medium supplemented with 10% horse serum and 5% yeast extract until mid-log phase as determined by acid-mediated shift of phenol red dye from red to orange, as previously described [2]. Cell density was measured and a mycoplasma concentration of 0.9×10^8^ to1.6×10^8^ cfu (colony forming unit) per milliliter, approximately mid-log phase, was used.

### Isolation of TX-114 phase proteins

*M. gallisepticum* strains R_low_ and R_high_ were grown to mid-log phase as previously described and pelleted by centrifuging at 10,000×g for 20 minutes. Pellets were washed twice with PBS and suspended in 750 µl of TS buffer (10 mM Tris, 150 mM NaCl, pH 7.5) containing 1 mM PMSF and 1% TX-114 and rocked for 30 minutes at 4°C, followed by centrifugation at 10,000×g at 4°C for 5 minutes. The soluble phase was transferred to a new tube and incubated at 37°C with rocking for 10 minutes and then centrifuged for 5 minutes at room temperature to separate the aqueous and detergent phases. 500 µl of TS buffer containing 1 mM PMSF and 1% TX114 was added to the detergent phase obtained in the previous step and thoroughly mixed and incubated for 15 min at 4°C followed by 10 min at 37°C with rocking followed by centrifugation for 5 minutes (at 10,000×g) at room temperature. The detergent phase was collected and the previous step was repeated to obtain the final detergent phase. The LAMPs were precipitated overnight at −20°C with 2 volumes of methanol and centrifuged at 15,000×g for 20 minutes. Precipitated protein was suspended in PBS by sonication for 30 seconds at output 5 using Biosonik ultrasonic disintegrator (Bronwill Scientific; Rochester, NY). Protein concentration was determined by Quick Start Bradford protein assay kit (Bio Rad; Hercules, CA) according to manufacturer's instructions.

### Cell culture and exposure/stimulation

A primary chicken tracheal epithelial cell culture was established based upon two previously described protocols [62], [63]. Five-week-old female specific pathogen free chicken tracheae were obtained from SPAFAS (Charles River Laboratories; Mansfield, CT) in sterile PBS (phosphate-buffered saline) containing 1X penicillin-streptomycin to inhibit bacterial and fungal growth. The tracheae were rinsed twice in DMEM (Dulbecco's modified eagles medium) (Gibco, Life technologies; Grand Island, NY) under sterile conditions. After removal of the surrounding adipose and muscular tissues, tracheae were cut into 2 cm pieces and incised vertically. They were then twice rinsed for 5 minutes in PBS/DTT (Dithiothreitol) (Sigma Aldrich; St. Louis, Missouri) (0.0385 g DTT- in 50 ml 1X PBS) to remove non-adherent mucus. The pieces were then rinsed twice with PBS and DMEM. To dissociate the epithelial cells from the underlying connective tissue, about 3–4 tracheal pieces were placed in a T25 flask containing 25 ml dissociation solution [DMEM 50 ml, protease type XIV 0.14 g (Sigma Aldrich), DNAse 0.01 g (Sigma Aldrich) and antibiotic/antimycotic 1X (Gibco, Life technologies)]. The flasks were gently shaken for 15 minutes at 37°C to slightly loosen the cells. 10% FBS (Gibco, Life technologies) was added to block protease activity after incubation. The tracheae were washed twice with fresh DMEM. The luminal surface of tracheae was scraped gently with a sterile scalpel in a petri dish containing fresh DMEM to obtain the epithelial cells. Cells were centrifuged at 1,250 rpm for 5 minutes to remove media. The epithelial cells were placed in 10 ml dissociation solution for 10 minutes at 37°C and then pipetted up and down several times to dissociate clumped cells. 10% FBS was added to the dissociation solution to stop the reaction. Cells were centrifuged at 1,250 rpm for 5 min to remove residual enzymes and washed twice with PBS. Cells were suspended in DMEM supplemented with 10% FBS and plated in a T75 flask for 4 hours to allow adherent cells (primarily fibroblasts and macrophages) to attach. The unattached cells were collected and precipitated by centrifugation at 1,250 rpm for 5 minutes and suspended in ATE medium [DMEM F-12+ Glutamax, (Gibco, Life technologies) 10% FBS (Gibco, Life technologies), 10% chick embryo extract (US Biologicals; Salem, MA), 1X MEM non-essential amino acids (NEAA) (Gibco, Life technologies), 1X antibiotic/antimycotic (Gibco, Life technologies)], counted on a hemocytometer and plated on 5% matrigel (BD Biosciences; San Jose, CA) coated T12.5 flasks. Exposure studies were done at 96 hours post-plating when the flasks reached 70–80% confluence, at which time they were exposed to either 5×10^8^ cfu (roughly equal to 500 multiplicity of infection (MOI) live strain R_low,_ R_high_ as previously described [64] or LAMPs isolated from each strain at a concentration of 5 µg/mL. This concentration was based on preliminary studies where 5 µg/mL and 50 µg/mL LAMPs showed comparable potency (based on changes in gene regulation) and roughly equivalent to 500 MOI (5×10^8^ cfu) live mycoplasma. All experiments were done with 6 replicates each for 1.5, 6, and 24 hours.

### Whole tracheal exposure

Tracheae from 5-week-old female specific-pathogen free white leghorn chickens were obtained from SPAFAS (Charles River Laboratories; Mansfied, CT) in sterile PBS containing 1X penicillin-streptomycin to inhibit bacterial and fungal growth. Surrounding adipose tissues were removed and tracheae were cut into 0.5 inch pieces by vertical incision and rinsed 3 times with PBS and DMEM under sterile conditions. The tracheae were exposed to either 10^9^ cfu *M. gallisepticum* strains R_low_ and R_high_ or 10 µg/mL LAMPs from either strain for 1.5 or 6 hours in ATE medium at 37°C, 5% CO_2_. After exposure, tracheae were digested in dissociation solution (described previously) for 15 minutes at 37°C and immediately placed on ice. Epithelial cells from the luminal surface of tracheal pieces were lightly scraped using a sterile scalpel. Epithelial cells were then preserved in RNA later (Ambion, Life Technologies; Grand Island, NY) at 4°C for future RNA isolation (detailed below). All exposures were done with 6 replicates each for 1.5, and 6 hours.

### Immunocytostaining

Tracheal epithelial cells and DF-1(Chicken embryonic fibroblast) cells grown on coverslips were fixed with 10% formalin and permeablized with 0.25% Triton X-100. Immunocytochemistry was performed using primary monoclonal anti-vimentin antibody (Sigma-aldrich) and anti E-cadherin antibody (Millipore; Billerica, MA) at a concentration of 1∶200. Fluorescence-tagged secondary antibodies used were goat anti mouse IgG FITC (Sigma-aldrich) for visualization of vimentin and Alexa Fluor 546 Goat Anti-Rabbit IgG (H+L) (Life Technologies; Grand Island, NY) for visualization of E-cadherin at a 1∶250 dilution. Vectashied HardSet mounting medium containing DAPI (4′, 6-Diamidino-2-phenylindole) (Vector Laboratories; Burlingame, CA) was used to mount cells on a slide for imaging. Images of immunostaining were captured using a Nikon A1R Spectral Confocal Microscope (Nikon Instruments Inc.; Melville, NY).

### Signaling inhibitors

Tracheal epithelial cells were incubated with either 3 µg/mL R_low_ LAMPs or 5 µg/mL R_high_ LAMPs in the presence or absence of signaling inhibitors. TLR-2/4 signaling inhibitor OxPAPC (oxidated 1-palmitoyl-2-arachidonyl-sn- glycero-3-phosphorylcholine), TLR-4 inhibitor CLI-095, and NF-κB inhibitor Celastrol were purchased from Invivogen (Invivogen; San Diego, CA). Cells were co-incubated with either 10 µg/mL or 30 µg/mL OxPAPC and LAMPs for 6 hours, in accordance with the manufacturer's instruction. Cells were pre-incubated with 1 µg/mL CLI-095 for 6 hours before exposure to LAMPs and then further incubated for 6 hours after LAMP exposure. Pre-incubation of cells with 5 µM Celastrol for 30 minutes was done before LAMP exposure for 6 hours. All experiments were performed with 6 replicates each.

### RNA isolation and cDNA synthesis

Total RNA was extracted from cells using TRIzol reagent (Life Technologies) and purified using RNeasy mini columns according to the manufacturer's instructions (Qiagen; Valencia, CA). On-column DNase digestion was done using RNase free DNase (Qiagen;). RNA quality and quantity was assessed using the Agilent 2100 Bioanalyzer with the RNA pico kit (Agilent technologies; Mendon, MA). All RNA samples had a RNA integrity number of 8 or more. 500 ng of RNA from individual samples was reverse transcribed using iScript reverse transcription supermix for RT-qPCR (Bio Rad; Hercules, CA) according to the manufacturer's recommendation, and 2 µg RNA was reverse transcribed for PCR reaction to assess epithelial-specific gene expression.

### Microarray

Agilent chicken gene expression microarray slides were utilized (Agilent technologies). Total RNA (50 ng) was utilized as starting material for microarray hybridization. Four biological replicates for each of five exposure conditions utilized were: R_high_, R_low_, R_high_ LAMPs, R_low_ LAMPs and media control. Dye swap technical replicates were created for each biological replicate totaling eight replicates for each exposure condition. The Agilent two-color microarray-based gene expression analysis protocol version 6.5 (http://www.genome.duke.edu/cores/microarray/services/agilent-microarrays/documents/LIQA_G4140-90050_GeneExpression_Two_Color_v6.5.pdf) was followed. All incubations were performed utilizing the Applied Biosystem Geneamp PCR system 9700 (Life Technologies). The Agilent two-color low-input quick amp labeling kit (Agilent technologies) was utilized for cDNA synthesis, *in-vitro* amplification and labeling of nucleic acids. Purification of labeled cRNA was performed with the Qiagen RNeasy mini kit). Purified cRNA was fragmented using the Agilent gene expression hybridization kit (Agilent technologies). Microarray slides and hybridization gasket were assembled in a hybridization chamber (Agilent technologies). Samples were placed in a rotating hybridization oven set to 10 rpm at 65°C for 17 hours. Microarray slides were scanned using a GenePix 4000B laser scanner (Molecular Devices; Sunnyvale, CA) following the instructions in the GenePixPro 7 user guide. Feature extraction was performed utilizing GenePixPro 7 software (Molecular Devices). Microarray images were visually inspected for quality control of features (spots on microarray slide that have spotted oligonucleotide probes). Background fluorescence of each feature was calculated as the mean of the five closest negative control features. Each channel's background-corrected median fluorescence value was used in an intensity-based analysis utilizing Agilent GeneSpring (v12.5) software (Agilent technologies). Quantile normalization was performed on all the background-corrected intensity values and samples were clustered according to their original exposure group. Features were removed if they were at saturating intensity or flagged “bad” in the aforementioned visual inspection. Genes were removed from downstream analysis if <80% of the features representing a gene were already excluded following preprocessing of data. Genes with duplicate or triplicate features on the microarray were grouped and their mean values were used for a gene-level analysis. Quantile normalization was then performed on the gene-level *in-silico* experiment. All microarray datasets have been deposited into Gene Expression Omnibus database repository, accession number GSE61520 (http://www.ncbi.nlm.nih.gov/geo/query/acc.cgi?acc=GSE61520).

### Polymerase Chain Reaction

PCR reactions were performed using cDNA to identify expression of epithelial cell-specific genes in tracheal epithelial cell cultures using GoTaq Green master mix (Promega; Madison, WI). Cycle conditions were as follows: 94°C for 3 minutes, 94°C for 30 seconds, 58°C for 30 seconds, 72°C for 1 minute. Step 2 to 4 was repeated 30 times. Final extension was performed at 72°C for 10 minutes. Primers for the epithelial cells specific genes are listed on **Table 1**.

**Table 1:** Epithelial gene specific primers.

Gene ID	Primer Name	Sequence 5′-3′
395209	Retinoic acid responder Forward	ACA TCA ACT CCC ACG AGG CGT CC
	Retinoic acid responder Reverse	ACT GCT GCC AAC AAT GGC CAA GC
408039	Keratin 14 Forward	CAC TGC CAG CCC GCT GTG CT
	Keratin 14 Reverse	ACC TTG TCC AGG TAG GCG GCC
407779	Keratin 5 Forward	TGC TGC TTT CCT GCT GCT CAG C
	Keratin 5 Reverse	ACG GTC ACT TCA TGG ATG CCA CC
414833	Cytochrome P-450 2C45 Forward	CCA CGT GGG AGA TGT TGC TCC TG
	Cytochrome P-450 2C45 Reverse	TGG CAG CAA ACT CAT CCG CAC G

### Real time quantitative PCR (RT-qPCR)

Primers specific for chicken genes were designed using Primer 3 input version 0.4.0 (http://frodo.wi.mit.edu/) or as described in Mohammed et al [12] (**Table 2**). RT-qPCR was performed using 1 µl of cDNA from the reverse transcription reaction using iTaq universal SYBR green supermix (Bio Rad). Amplification was performed using an Applied Biosystem 7900 HT (Life Technologies) by incubating samples at 50°C for 2 minutes, 95°C for 1 min, followed by 40 cycles of 95°C for 15 seconds and 58–60°C (depending on Tm values of specific primers) for 60 seconds. Melt curve analysis was performed to confirm that a single, product-specific amplification had occurred. A stepwise temperature gradient was used (65–95°C) with 0.5°C increments and 2 sec/step. Amplicon sizes were confirmed by agarose gel electrophoresis. The reference gene for all real time reactions was GAPDH. Absolute fold change compared to the media controls were calculated using the Ct values based upon the following equation:
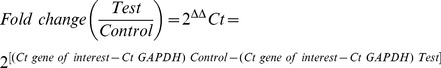



**Table 2:** Gene specific primers for RT-qPCR.

Primer Name	Sequence 5′-3′
GAPDH Forward	ATT CTA CAC ACG GAC ACT TCA
GAPDH Reverse	CAC CAG TGG ACT CCA CAA CAT A
IL-12p40 Forward	TGAAGGAGTTCCCAGATGC
IL-12p40 Reverse	CGTCTTGCTTGGCTCTTTATA
IL-1β Forward	GCT GGA ACT GGG CAG AT
IL-1β Reverse	GGT AGA AGA TGA AGC GGG TC
IL-8 Forward	GTG CAT TAG CAC TCA TTC TAA GTT
IL-8 Reverse	GGC CAT AAG TGC CTT TAC G
IL-6 Forward	CCT GTT CGC CTT TCA GAC CTA
IL-6 Reverse	AGT CTG GGA TGA CCA CTT C
IL-10 Forward	AGAGATGCTGCGCTTCTACA
IL-10 Reverse	GCTTGATGGCTTTGCTCC
CCL-20 Forward	GCC AGA AGC TCA AGA GGA TG
CCL-20 Reverse	TCC AGA AGT TCA ACG GTT CC
NOS-2 Forward	TGA TCT TTG CTG CCA AAC AG
NOS-2 Reverse	TCC TCT GAG GGA AAA TGG TG
miRNA-146a Forward	GAGAACTGAATTCCATGGGTTG
miRNA-146a Reverse	TCCAAGCTGAAGAACTGAGC

Gel electrophoresis was performed using a 0.8% agarose gel to determine the product size for each gene.

### Statistical analysis

All statistical analyses were performed using the Statistical Analysis Software (SAS) program Version 9.2 (SAS Institute; Inc Cary, NC). Multiple pairwise comparisons of mRNA fold differences were analyzed using the mixed design analysis of variance (ANOVA) with repeated measures, with mRNA fold difference being the dependent variable and treatments or time being the independent variables. Post hoc mean comparison analyses were performed using least square means. Results were denoted as fold change ± SEM. Significant differences were denoted as * = P<0.05, ** = P<0.01, *** = P<0.001.

## Results

### Tracheal epithelial cell culture and immunocytostaining

**Figure 1:**
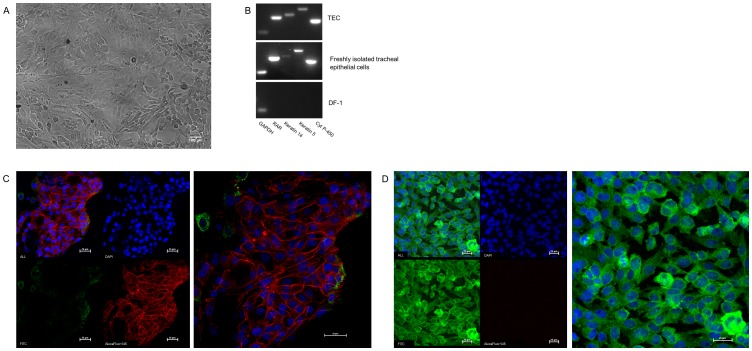
Primary chicken tracheal epithelial cell culture (TEC). Primary chicken tracheal epithelial cells were isolated and cultured as described in the Methods section. **1A:** Primary chicken tracheal epithelial cells at 100X magnification. **1B:** Confirmation of tracheal epithelial cell identity both *in vitro* and freshly isolated (*ex vivo*) from tracheae after *ex-vivo* exposure: PCR amplified epithelial cell specific genes from cDNA in agarose gel, compared to chicken embryonic fibroblast (DF-1) cells. **1C:** Tracheal epithelial cells stained for E-cadherin and Vimentin at (400X magnification). Left panel shows TECs at different filter setting Blue (DAPI) for nuclear staining, Green (FITC) for Vimentin and Red (AlexaFluor 546) for E-cadherin, right panel shows merged picture for all filters. **1D:** DF-1 fibroblast cells stained for E-cadherin and Vimentin at 400X magnification. Left panel shows DF-1 cells at different filter setting; Blue (DAPI) for nuclear staining, Green (FITC) for Vimentin and Red (AlexaFluor 546) for E-cadherin; right panel shows merged picture for all filters.

Primary tracheal epithelial cell cultures were established based on published methods [62], [63]. Confirmation was performed using PCR amplification of epithelial cell specific genes and morphological examination based on E-cadherin staining patterns. A chicken embryonic fibroblast cell line (DF-1) was used as a negative control. E-cadherin staining was observed only at the contact points between cells, which is typical of epithelial cell morphology. Very few cells in the TEC culture stained for vimentin, suggesting an extremely low level of fibroblast contamination. As a positive control, DF-1 chicken fibroblast cells stained positively for vimentin and not for E-cadherin (**Figure 1A–D** **, Figure S4**).

### Microarray analysis – global gene expression profile

**Figure 2:**
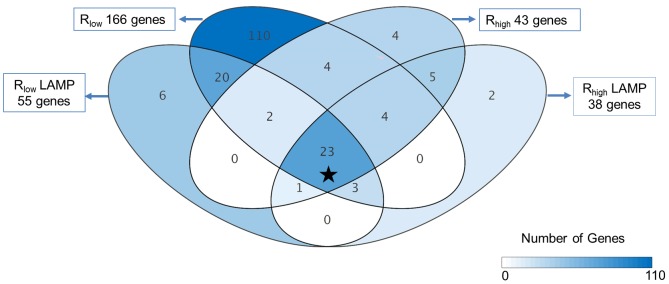
Distribution of differentially regulated genes in TECs. Differentially regulated genes (≥5 fold) in tracheal epithelial cell after exposure to live R_low_, R_high_ or LAMPs isolated from either strain 1.5 hours after exposure. The star (*) in the figure represent commonly up-regulated genes upon all four exposures, from which six follow up genes were chosen. n = 8 (4 biological replicates x2 dye swap technical replicates) for all microarray experiments.

Microarray analysis of chicken tracheal epithelial cells at 1.5 hours post-exposure identified a total of 166, 43, 55 and 38 genes differentially regulated ≥5-fold (p≤0.05) after exposure to either live R_low_ and R_high,_ or to 5 µg/mL R_low_ or R_high_ LAMPs respectively (**Figure 2**). 23 genes were commonly up-regulated ≥5-fold in TECs in all four exposure groups, which included many inflammatory chemokine and cytokine genes (**Figure 2** ; indicated by the asterisk*). Gene ontology analysis of commonly up-regulated genes (≥2-fold) identified categories such as immune system processes, signal transduction, regulation of apoptosis and stress response (**Table 3** **, Figure S1 and Table S1**). Exposure to viable R_low_ resulted in differential expression of 110 unique genes by a factor of ≥5- fold (p≤0.05) (**Table S2**) whereas only six or fewer genes were unique in TECs exposed to R_high_, R_low_ LAMPs or R_high_ LAMPs (**Figure 2**). Pathway analysis identified differential expression of genes in the TLR signaling pathway, with 12 out of 70 genes commonly up-regulated in all four exposure groups. Other pathways of note included the TNF-α/NF-κB signaling, apoptosis, and type II interferon pathways (**Table 4** **and Figure S3**).

**Table 3:** Genes of significant interest from microarray analysis.

Entrez ID	Gene Name	mRNA Fold Change in TECs exposed to:			
		R_low_	R_high_	R_low_ LAMP	R_high_ LAMP
404671	Interleukin 12B	207.80	71.80	194.67	78.21
395196	Interleukin 1, beta	9.89	7.86	6.75	5.65
395082	Chemokine (C-C motif) ligand 20	55.34	11.67	43.92	12.98
395337	Interleukin 6	42.33	30.57	27.31	20.95
396495	Interleukin 8	22.92	20.72	17.87	19.44
395807	Nitric oxide synthase 2, inducible	10.53	3.68	8.41	3.94
396451	Prostaglandin-endoperoxide synthase 2	12.98	10.65	13.13	9.76
374012	Baculoviral IAP repeat-containing 2	5.42	4.07	5.45	3.48
396384	Interferon regulatory factor 1 (IRF1)	5.66	5.48	4.73	5.07
396424	Plasminogen activator, urokinase	7.15	16.65	7.95	14.86
396033	Nuclear factor of kappa light polypeptide gene enhancer in B-cells 1	2.55	2.08	2.58	2.02
418404	Nuclear factor of kappa light polypeptide gene enhancer in B-cells inhibitor, zeta	10.24	17.96	12.14	15.32
396093	Nuclear factor of kappa light polypeptide gene enhancer in B-cells inhibitor, alpha	5.00	4.15	5.00	3.62
417465	Chemokine (C-C motif) ligand 5	2.63	2.65	2.38	2.65
396330	Interferon regulatory factor 7	6.57	5.21	5.44	4.22
421219	Toll-like receptor 15	2.75	2.53	3.69	2.11
396093	Nuclear factor of kappa light polypeptide gene enhancer in B-cells inhibitor, alpha	5.00	4.15	5.00	3.62
417247	Similar to TL1A; tumor necrosis factor (ligand) superfamily, member 15	4.22	14.47	4.08	10.33
423471	TNF receptor-associated factor 3	3.68	5.35	4.39	5.46
408036	Epiregulin	4.01	2.02	2.80	2.66
422219	Interleukin 13 receptor, alpha 2	5.40	3.25	4.57	3.64
421686	Interleukin 20 receptor, alpha	5.01	3.73	3.72	3.97
424704	Interleukin 23 receptor	2.34	2.41	2.48	4.44
422884	TNFAIP3 interacting protein 2	10.95	4.77	7.09	7.01
768950	CD80 molecule	2.96	4.77	2.44	4.02

Representative list of Table S1: mRNA fold changes ≥2 (p-value ≤0.05) in TECs exposed to R_low_, R_high,_ R_low_ LAMP and R_high_ LAMP compared to control.

**Table 4:** Pathway analysis of differentially regulated genes.

Pathway	No. of genes from different pathways up-regulated in TEC's exposed to:				Total no. of genes in pathway
	R_low_	R_high_	R_low_ LAMP	R_high_ LAMP	
Toll-like receptor signaling pathway	17	15	13	13	70
TNF-alpha NF-kB Signaling Pathway	10	9	11	10	157
Adipogenesis	14	7	4	4	100
Senescence and Autophagy	10	8	7	7	73
EGFR1 Signaling Pathway	8	4	5	2	148
MAPK signaling pathway	5	7	4	5	123
Apoptosis	5	6	5	6	65
Type II interferon signaling pathway	6	4	4	4	22

Analysis performed using GeneSpring (v12.5), within genes that are differentially expressed ≥2-fold (p-value ≤0.05).

### Differential gene expression in R_low_, R_low_ LAMP, R_high_ and R_high_ LAMP exposed TECs

**Figure 3:**
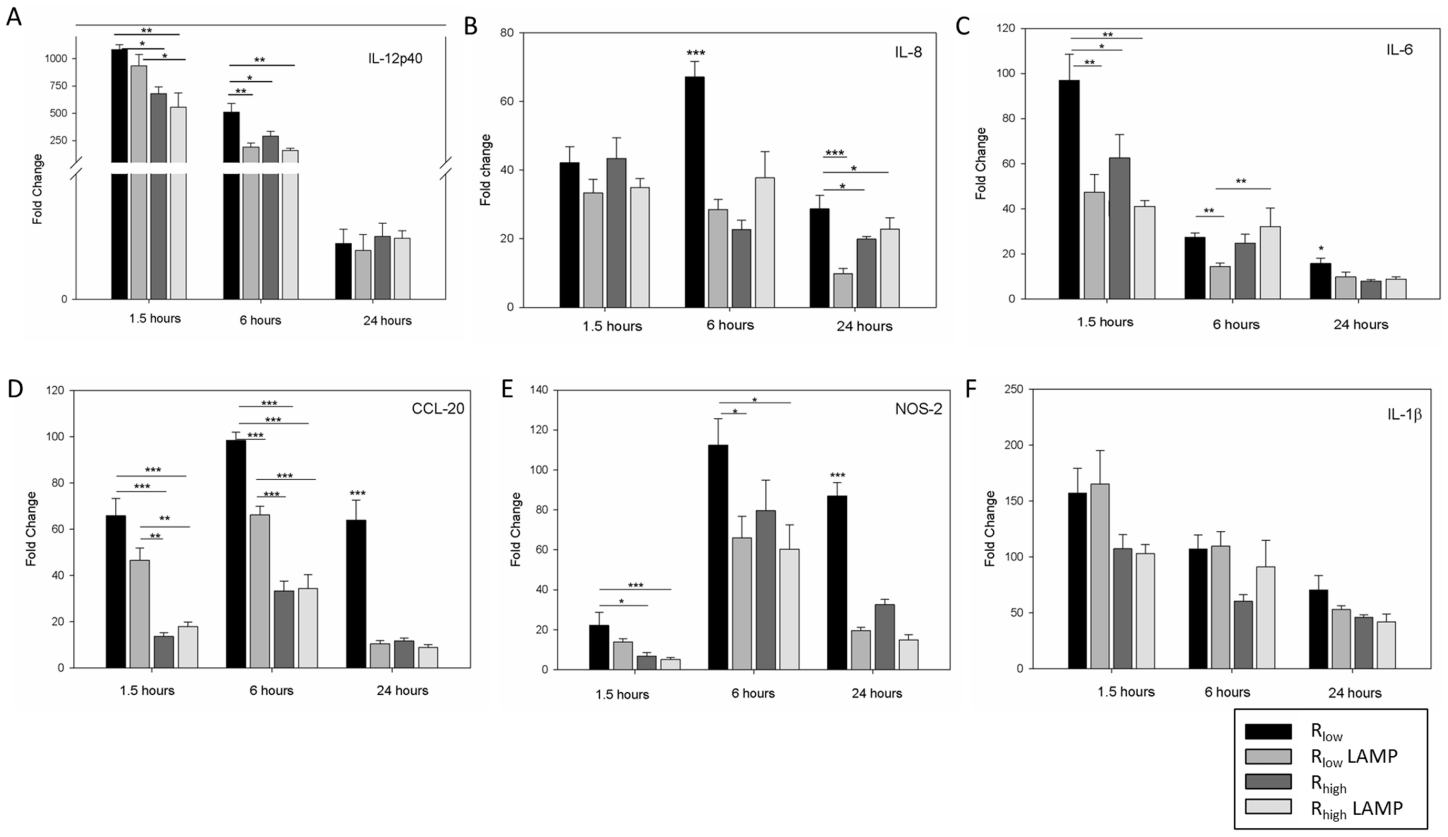
Differential gene expression in TECs post-exposure. mRNA fold difference in TECs exposed to R_low_, R_low_ LAMP, R_high_ or R_high_ LAMP at 1.5, 6 and 24 hours respectively. Samples normalized to housekeeping gene GAPDH and un-exposed TECs as control. n = 6 for all experiments. Results are denoted as fold change ± SEM with all control values set at 1. Significant differences denoted as * = P<0.05, ** = P<0.01, *** = P<0.001. **A:** IL-12p40 mRNA. **B:** IL-8 mRNA. **C:** IL-6 mRNA. **D:** CCL-20 mRNA. **E:** NOS-2 mRNA. **F:** IL-1β mRNA.

A concentration of 5 µg/mL LAMPs optimally induced the expression of IL-1β and CCL-20, which was roughly comparable to the up-regulation induced by 500 MOI (5×10^8^ cfu) live mycoplasma (data not shown). mRNA transcripts of six immune response associated genes up-regulated in TECs upon exposure to each of the four treatments were validated by RT-qPCR at 1.5, 6 and 24 hours. TECs' expression of IL-12p40, IL-1β and IL-6 peaked at 1.5 hours post-exposures in all four exposure groups and waned significantly thereafter in contrast to CCL-20 and nitric oxide synthase-2 (NOS-2) which peaked at 6 hours and returned to near baseline levels within 24 hours. IL-8 expression peaked at 6 hours in R_low_ exposed TECs, however R_high_ and R_low_ LAMP exposed TECs exhibited the highest level of IL-8 gene expression at 1.5 hours (p≤0.01 and p≤0.001) (**Figure S2A–D**). Five out of six genes (IL-6, IL-8, IL-12p40, CCL-20 and NOS-2) were expressed at significantly higher levels in R_low_ exposed TECs when compared to TECs exposed to R_high_ or LAMPs isolated from either strain, at one or more time point (**Figure 3A–E**). IL-1β gene expression in TECs did not significantly differ between any of the four exposures at any time point (**Figure 3F**). We also observed that R_low_ LAMP exposed TECs had a significantly higher level of the IL-12p40 gene expression when compared to TECs exposed to R_high_ LAMP at 1.5 hours (p≤0.05) (**Figure 3A**) and CCL-20 when compared to TECs exposed to R_high_ or R_high_ LAMP at 1.5 and 6 hours (p≤0.01 and p≤0.001) (**Figure 3D**).

### Differential gene expression in TECs by mycoplasma LAMPs in the presence of signaling inhibitors

**Figure 4:**
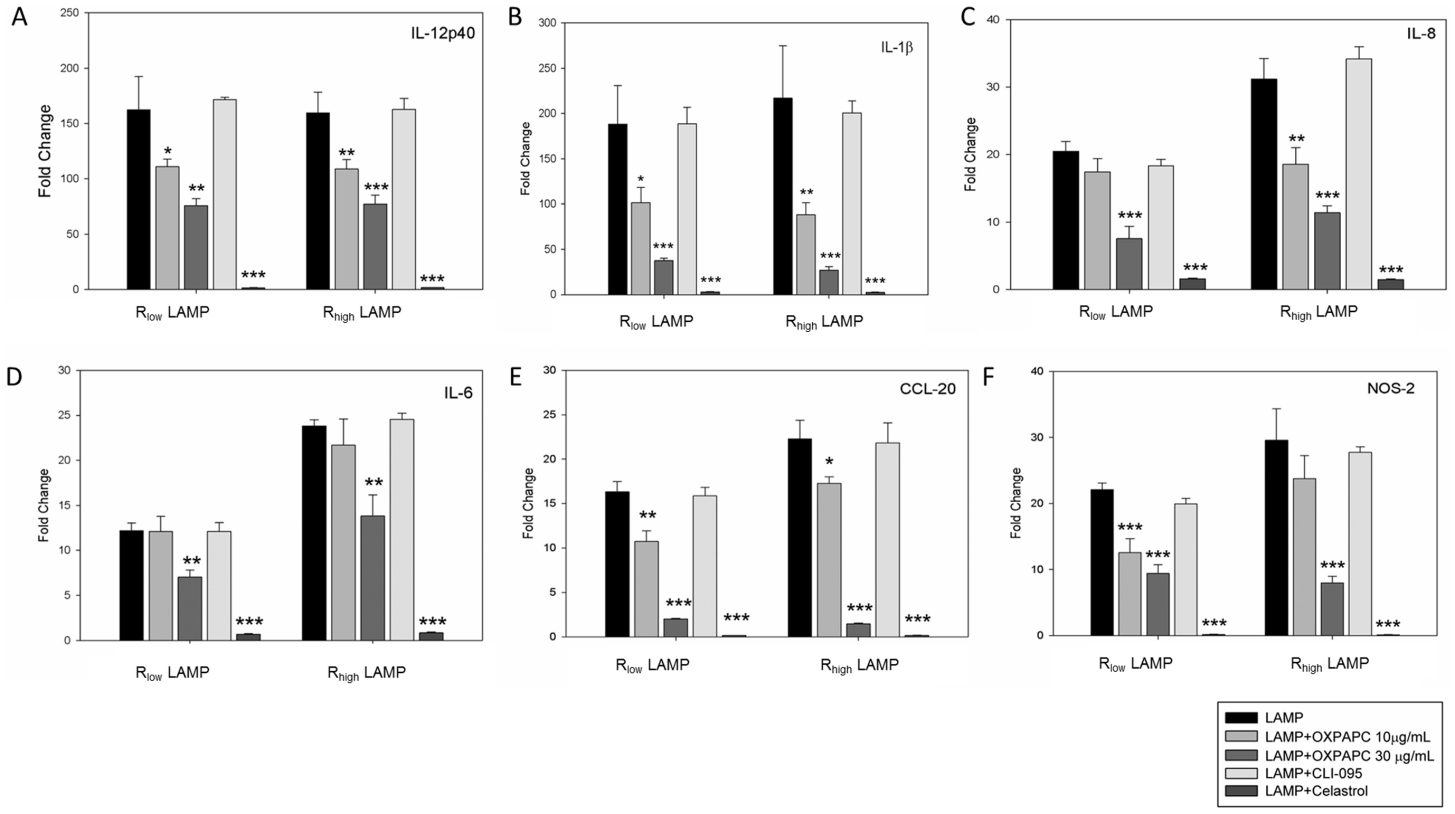
Differential gene expression in TECs exposed to LAMPs in the presence of signaling inhibitors. Epithelial cells were exposed to LAMPs isolated from R_low_ or R_high_ in the presence or absence of signaling inhibitors for 6 hours. Samples were normalized to the housekeeping gene GAPDH and un-exposed TECs served as control. n = 6 for all experiments. Results are denoted as fold change ± SEM with all control values set at 1. Significant differences denoted as * = P<0.05, ** = P<0.01, *** = P<0.001. **A**. IL-12p40. **B**. IL-1β. **C**. IL-8. **D**. IL-6. **E.** CCL-20. **F**. NOS-2.

OxPAPC, a TLR-2 and 4 signaling inhibitor, reduced expression of all 6 genes in a concentration dependent manner (**Figure 4A–F**). IL-12p40 gene expression was reduced by more than 2-fold in TECs exposed to LAMPs when incubated with OxPAPC at 30 µg/mL when compared to both R_low_ (P<0.05) and R_high_ LAMP (P<0.01) exposed TECs (**Figure 4A**). IL–1β and CCL-20 gene expression were reduced by more than 5-fold compared to TECs exposed to R_low_ (P<0.001) or R_high_ (P<0.001) LAMPs in the absence of the inhibitor (**Figure 4B, E**). IL-8 (P<0.001), IL-6 (P<0.01) and NOS-2 (P<0.001) gene expression was also significantly reduced by approximately 2–3 fold (**Figure 4C, D, and F**). No difference was observed upon exposure to CLI-095, a selective TLR-4 inhibitor, for any of the genes analyzed in this study, supporting the hypothesis that *M. gallisepticum* LAMPs signal via TLR-2 and not TLR-4 (**Figure 4A–F**). In the presence of Celastrol, an NF-κB inhibitor, cell signaling was abolished entirely (comparable to control levels) in TECs exposed to any of the four treatments (P<0.001 for all six genes analyzed) (**Figure 4A–F**).

### Comparison of gene regulation in R_low_, R_low_ LAMP, R_high_ and R_high_ LAMP exposed tracheal epithelial cells *ex vivo*

**Figure 5:**
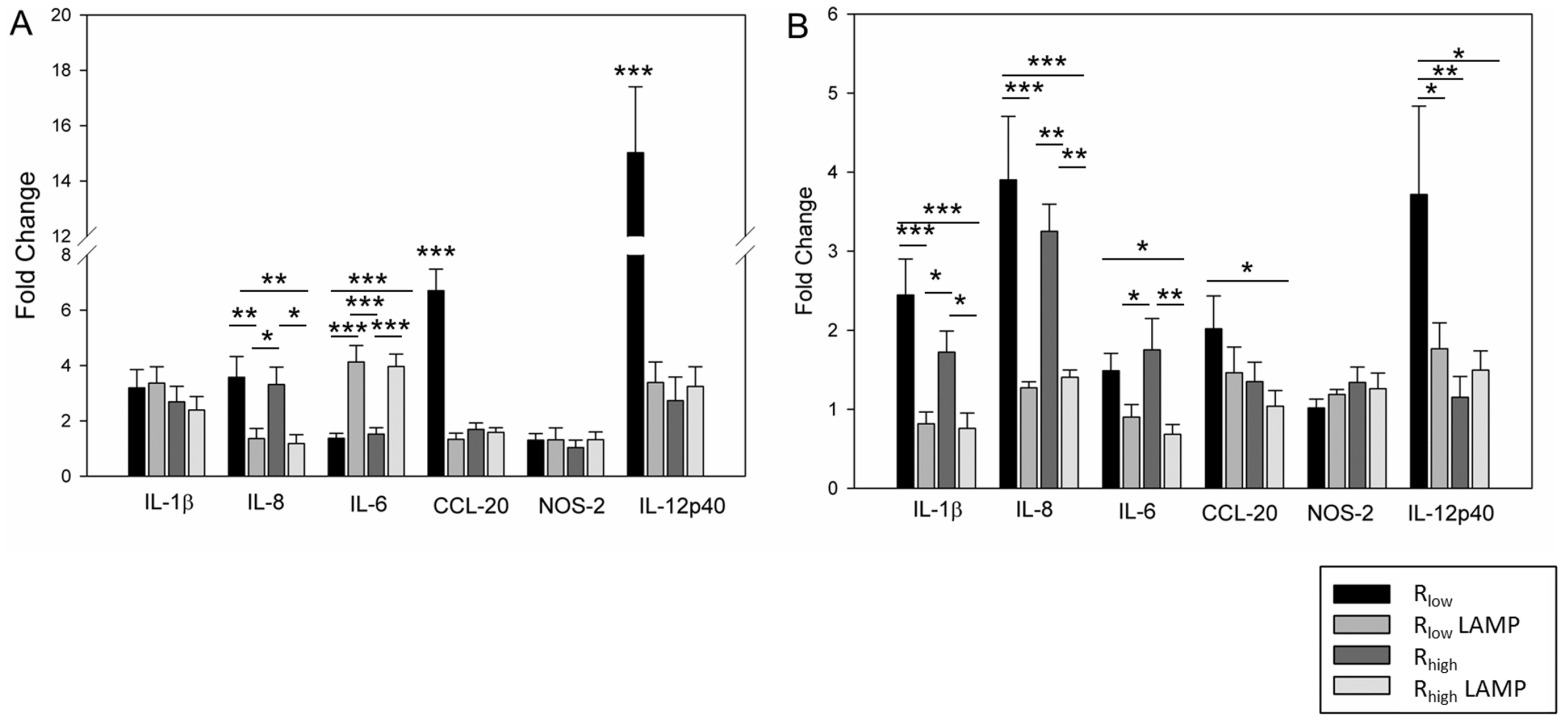
Differential gene expression in tracheal epithelial cells after *ex-vivo* exposure to LAMPs. Comparison of mRNA fold difference in tracheal epithelial cells from tracheal explant exposed to R_low_, R_low_ LAMP, R_high_ or R_high_ LAMP at 1.5 and 6 hours respectively. Samples normalized to housekeeping gene GAPDH and un-exposed tracheae as control. n = 6 for all experiments. Results are denoted as fold change ± SEM with all control values set at 1. Significant differences denoted as * = P<0.05, ** = P<0.01, *** = P<0.001. **A:** mRNA fold difference of all genes at 1.5 hours. **B:** mRNA fold difference of all genes at 6 hours.

mRNA transcripts of all six genes were also up-regulated in tracheal epithelial cells isolated from whole tracheal tissues exposed *ex vivo* to live R_low_, R_high_ or the LAMPs isolated therefrom. Most genes followed a similar pattern of expression as seen in the *in vitro* experiments. IL-12p40 was expressed at a significantly higher level in R_low_ exposed trachea at both time points compared to others, and CCL-20 was significantly higher in R_low_ exposed trachea than all others at 1.5 hours (p≤0.001) (**Figure 5A, 5B**). IL-8 expression was higher in both R_low_ and R_high_ exposed trachea when compared to the LAMP exposed tracheae at both time points but IL-6 was found to be higher in the LAMP-exposed tissues (p≤0.001) at 1.5 hours and lower at 6 hours (p≤0.01, p≤0.05) when compared to tracheae exposed to live R_low_ or R_high_. Although IL–1β expression did not differ between any exposure groups at 1.5 hours, at 6 hours tracheae exposed to the live mycoplasma were found to express IL–1β at a higher level than either LAMP-exposed tracheae (p≤0.001 for R_low_, p≤0.05 for R_high_). NOS-2 expression did not differ between any exposure groups at any time point (**Figure 5A, 5B**).

### Micro RNA and IL-10 gene expression

**Figure 6:**
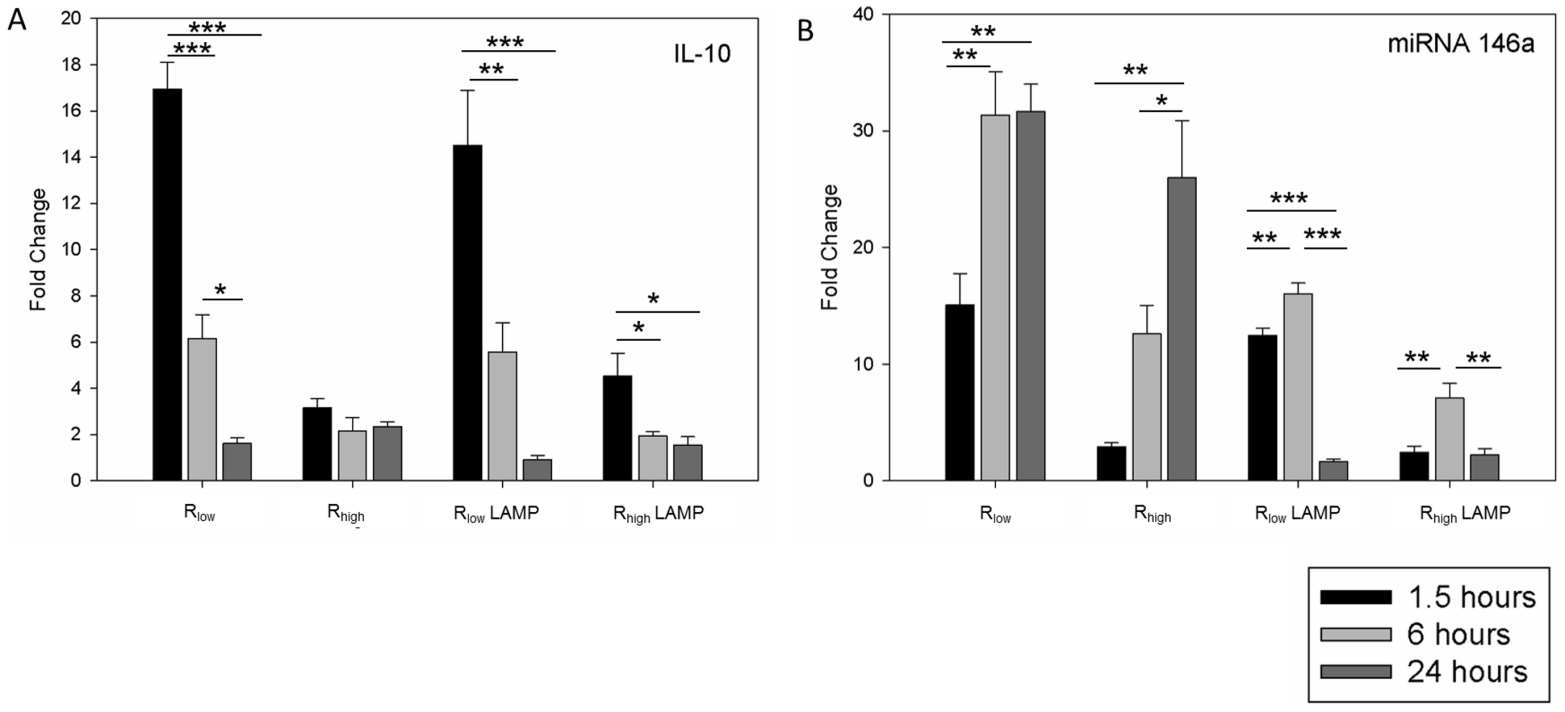
RT-qPCR analysis of miRNA and IL-10 differential expression in TECs. Epithelial cells were exposed to R_low_, R_low_ LAMP, R_high_ or R_high_ LAMP at 1.5, 6 and 24 hours respectively. Samples were normalized to housekeeping gene GAPDH and un-exposed TECs as control. n = 6 for all experiments. Results are denoted as fold change ± SEM with all control values set at 1. Significant differences denoted as * = P<0.05, ** = P<0.01, *** = P<0.001. **A:** mRNA fold difference of IL-10 in TECs at all three time points post exposure. **B:** mRNA fold difference of miRNA-146a in TECs at all three time points post exposure.

Expression analysis of four miRNA genes including mir-21, mir-146a, mir-146b, and mir-146c1, as well as IL-10 was assessed in TECs exposed to either LAMPs or live organisms. IL-10 gene expression in all TECs peaked at 1.5 hours, concomitant with the pro-inflammatory genes (**Figure 6A**). Expression analysis of miRNAs showed that miRNA146a increased over time in TECs exposed to live mycoplasmas; however in TECs exposed to the LAMPs from both strains, miRNA146a expression peaked at 6 hours (**Figure 6B**). Expression of miRNA21, miRNA146b and miRNA146c1 did not differ from media controls at any time point in any exposure group (data not shown).

## Discussion

Bacterial cell envelope components such as LPS, lipotechoic acid, peptidoglycan, flagella and lipoproteins are well characterized PAMPs that interact with host cell pattern recognition receptors such as TLRs, thereby contributing in part to the inflammation that ensues post-infection [20], [43], [65]. With the exception of lipoproteins, *M. gallisepticum* is devoid of these PAMPs, yet is able to initiate a robust inflammatory response marked by infiltration of leukocytes to the submucosa, often in absence of tissue invasion [2], [12], [13], [66], [67]. Mycoplasma lipoproteins are well known for their pro-inflammatory properties, initiated upon TLR ligation and NF-κB activation [34], [48]–[51], [55], [56], [68]. However, the vast majority of studies examining these effects have focused on monocytes/macrophages maintained in culture rather than epithelial cells, the primary site of mycoplasma attachment and colonization [11], [69].

Previous studies from our laboratory in which live birds were exposed to *M. gallisepticum* intra-tracheally reported alterations in inflammatory gene expression in whole tracheal tissue [12]. The current study is novel in that it reports interaction of *M. gallisepticum* lipid associated membrane proteins, a mixture of lipoproteins, specifically with host airway epithelial cells.

A study by Walter et al (2001) reported that IL-12p40 was expressed by airway epithelial cells during viral tracheobronchitis [70], [71]. We also observed IL-12p40 mRNA to be significantly up-regulated both in TECs and epithelial cells from tracheae upon exposure to live mycoplasma or LAMPs within 1.5 hours of exposure. But this signal waned rapidly thereafter, suggesting that IL-12p40 acts as an early, but not sustained, inflammatory event. IL-1β and IL-6 mRNA expression were also up-regulated and followed similar kinetics to that observed with IL-12p40. mRNA expression of two important chemokines, CCL-20 and IL-8 were also significantly up-regulated in epithelial cells *in-vitro* and *ex-vivo* after exposure to LAMPs or live organisms, as was NOS-2. These molecules are known for their ability to chemo-attract and activate leukocytes at the site of infection [72]–[77]. Several other inflammation associated genes, including TLR-15, were up-regulated to a lesser degree in TECs in all four exposure groups (**Table 3**, RT-qPCR data not shown). This cytokine and chemokine expression profile is in keeping with earlier studies that reported *M. pneumoniae* and *M. genitalium* mediated production of IL-8, TNF-α, IL-1β, IL-6 IL-7, MCP-1 and GM-CSF from epithelial cells [25]–[28].

A previously published study from our laboratory however, reported down-regulation of IL-12p40, IL-8, IL–1β, and CCL-20 mRNA in tracheal tissues at day-1 post-infection, whereas chemokines like MIP-1β, CXCL-13, RANTES and lymphotactin were found to be up-regulated [12]. As this later set of chemokines are known to be produced primarily by macrophages, lymphocytes and NK cells [14]–[19], types of cells not found in large numbers in normal tracheal mucosa, we believe transient but robust expression of chemokines and cytokines like IL-12p40, IL-8, IL-6, IL–1β, and CCL-20 by tracheal epithelial cells may be responsible for initially attracting the inflammatory cells into the tracheal submucosa. However these signals appear to be transient and subsequent signaling events appear to involve a unique set of inflammatory genes not observed in epithelial cells.

In the current study we also observed that R_low_ was by far the most potent stimulus for initiating differential gene expression by epithelial cells when compared to live R_high_ or LAMPs from either strain. TECs exposed to R_low_ not only up-regulated inflammatory genes to a significantly higher extent, but up-regulated more than a hundred additional unique genes by ≥5 fold. The R_high_ strain is non-virulent as it lacks several virulence determinant proteins of R_low_, *especially* GapA and CrmA [7], [78] two significant cytadhesion-associated proteins homologous to P1 and P40/P90 of *M. pneumoniae* [78]–[84]. The significantly diminished cytadhesion capability of R_high_ [7], [82], [85], [86], may explain the reduced differential gene expression induced by this strain. Subtle differences in gene expression observed between the LAMPs prepared from R_low_ and R_high_ may also be explained by the differences identified by Szczepanek et al. involving 29 mutations in the variable GAA repeat region associated with phase variable expression of vlhA genes between these two strains [86].

When TECs were exposed to LAMPs in the presence of OxPAPC, a competitive inhibitor of lipoprotein and LPS mediated signaling via TLR-2 and TLR-4 respectively [87], expression of all six genes was significantly reduced. However in the presence of CLI-095, a selective TLR-4 inhibitor, no changes in gene expression were observed. When TECs were exposed to Celastrol, an NF-κB inhibitor [88], LAMP-mediated gene expression was completely abrogated. This observation is in keeping with previous studies from other mycoplasma species in which LAMP-induced inflammation was mediated upon TLR-2 ligation and activation of NF-κB [28], [34], [49]–[51], [53], [55], [56]. Moreover, these observations apply to early time-points post-exposure, in a highly relevant cell population.

The kinetics of inflammatory gene expression in the current study was found to be rapid, peaking at either 1.5 hours or 6 hours, and then waning by 24 hours. As modulation of host responses is often accompanied by concurrent pro- and anti-inflammatory mechanisms [89], we hypothesized that a compensatory, homeostatic mechanism may be working in concert with the inflammatory response. Upon NF-κB activation, certain anti-inflammatory genes and micro RNA's are expressed that participate in the homeostatic regulation of inflammatory responses. For example, miRNA-146a, which is induced by LPS via NF-κB activation, down-regulates IRAK-1 and TRAF-6 and in turn suppresses further activation of NF-κB [90]. miRNA146a also negatively regulate TLR-2 signaling [91]. miRNA-21, on the other hand can promote IL-10 production by regulating PDCD4 (programmed cell death 4), an inhibitor of IL-10 production [90]. We observed miRNA-146a expression to be increasingly up-regulated until 24 hours in TECs exposed to either R_low_ or R_high_ in contrast to the pattern observed upon exposure to LAMPs, which peaked at 6 hours and waned thereafter, likely due to the lack of LAMP re-stimulation [91]. No difference in expression was observed for miRNA146b, miRNA146c1 or miRNA21. Conversely, the anti-inflammatory cytokine IL-10 showed no reciprocal relationship to pro-inflammatory gene expression, but was significantly up-regulated by the epithelial cells initially upon exposure to live R_low_ and the LAMPs (but not R_high_). IL-10 is known to selectively inhibit nuclear localization of NF-κB by blocking IκB kinase activity and inducing nuclear translocation and DNA-binding of the repressive p50–p50 homodimer [92], [93]. Therefore, miRNA-146a and in part IL-10 may play a role in regulating the over-exuberant pro-inflammatory response observed during *M.gallisepticum* infection.

Taken together our data suggest that *M. gallisepticum* LAMPs have potent inflammatory properties and can mediate changes in gene expression in chicken tracheal epithelial cells almost immediately upon exposure. However, the response appears to be transient in nature likely due to host compensatory mechanisms. Thus, continuous LAMP-mediated stimulation by adherent, replicating mycoplasma may be necessary to sustain the response. Studies using chemical inhibitors of specific signaling pathways indicated that mycoplasma LAMPs ligate TLR2 on TECs and activate NF-κB resulting in downstream expression of several pro-inflammatory chemokines and cytokines. Among the up-regulated genes are chemokines and cytokines known for leukocyte chemo-attraction and activation, consistent with the immunopathology associated with infection. Our data also support the notion that the virulent R_low_ strain possesses additional mechanisms of initiating inflammatory responses in tracheal epithelial cells beyond that mediated by LAMPs alone.

## Supporting Information

Figure S1 **Distribution of differentially regulated genes in TECs.** Differentially regulated genes (≥2 fold) in tracheal epithelial cells after exposure to live R_low_, R_high_ or LAMPs isolated from either strain for 1.5 hours. n = 8 (4 biological replicates x2 dye swap technical replicates) for all microarray experiments.
(TIF)

Figure S2 **Kinetic analysis of differentially regulated genes encoding inflammatory chemokines and cytokines.** Differential gene expression in TECs exposed to R_low_, R_low_ LAMP, R_high_ or R_high_ LAMP at 1.5, 6 and 24 hours respectively. Samples normalized to housekeeping gene GAPDH and un-exposed TECs as control. n = 6 for all experiments. Results are denoted as fold change ± SEM with all control values set at 1. Significant differences denoted as * = P<0.05, ** = P<0.01, *** = P<0.001. **A**: mRNA fold difference in R_low_ exposed cells **B**: mRNA fold difference in R_high_ exposed cells. **C**: mRNA fold difference in R_low_ LAMP exposed cells. **D**: mRNA fold difference in R_high_ LAMP exposed cells.
(TIF)

Figure S3 **TLR- Signaling pathway.** Toll like receptor signaling network: Common genes up-regulated in TECs exposed to R_low_, R_high_, R_low_ LAMP or R_high_ LAMP are depicted in yellow boxes.
(TIF)

Figure S4 **Original agarose gels photos of amplified products of epithelial cell specific genes.** **1A:** Confirmation of tracheal epithelial cells in culture *in vitro* using amplification of epithelial cell specific genes compared to chicken embryonic fibroblast (DF-1) cells. **1B:** Confirmation of epithelial cell specific genes in freshly isolated epithelial cells from tracheae after *ex-vivo* exposure.
(TIF)

Table S1 **Differentially expressed genes (≥2-fold, p-value ≤0.05).** Genes commonly up-regulated in TECs exposed to R_low_, R_high,_ R_low_ lipoprotein and R_high_ lipoprotein: analyzed for gene ontology hierarchal clustering (Excludes unknown genes listed as finished cDNA clones).
(DOCX)

Table S2 **Differentially expressed unique genes in R_low_ exposed TECs (≥5-fold, p-value ≤0.05) (Excludes unknown genes listed as finished cDNA clones).**
(DOCX)
