# Supplementary figures and images for: Mycoplasma gallisepticum Lipid Associated Membrane Proteins Up-regulate Inflammatory Genes in Chicken Tracheal Epithelial Cells via TLR-2 Ligation through an NF-κB Dependent Pathway
Source: PLoS One. 2014 Nov 17;9(11):e112796. doi: 10.1371/journal.pone.0112796 (PMC4234737; doi:10.1371/journal.pone.0112796)

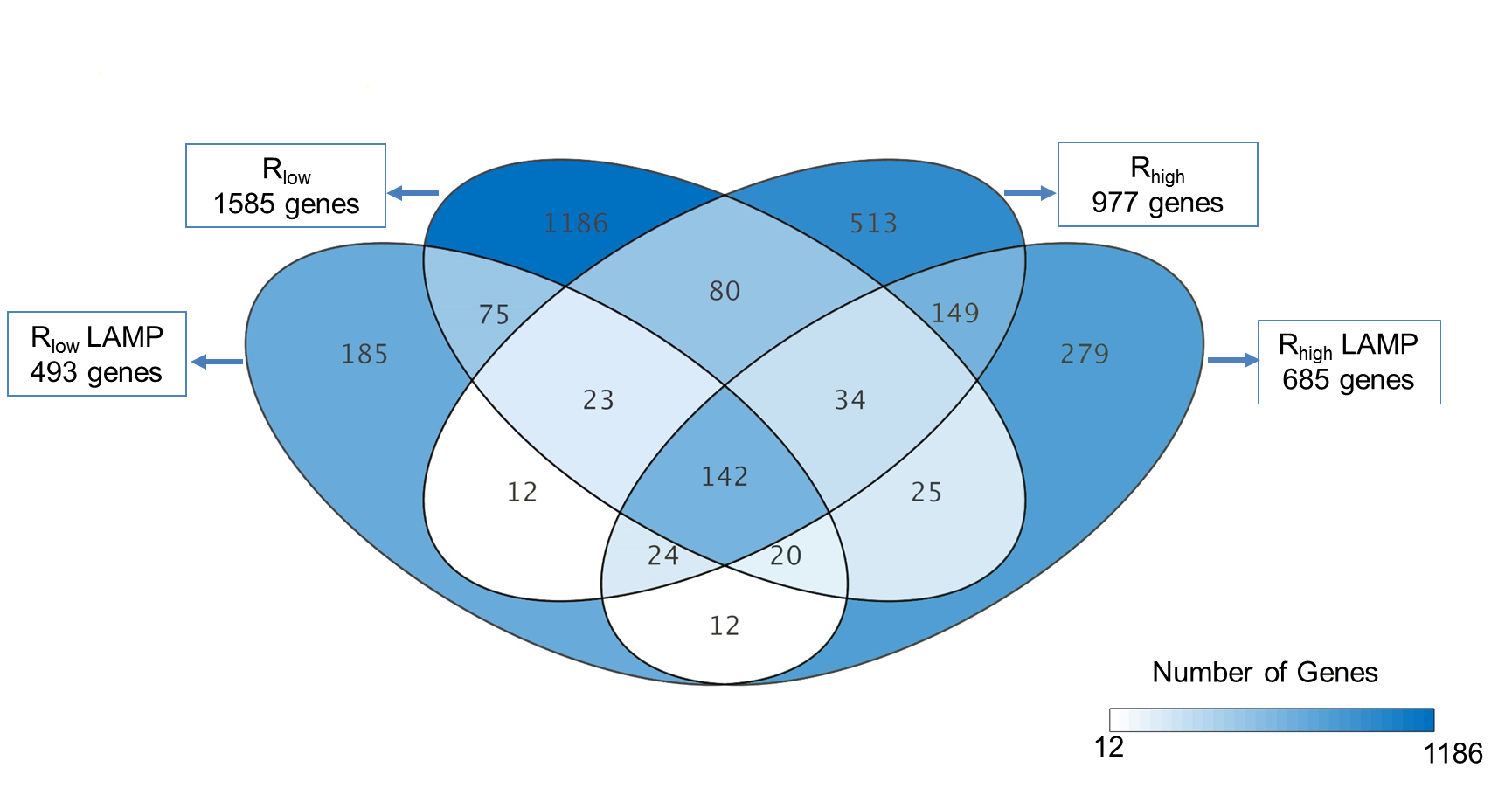

Supplement: Figure S1 — Distribution of differentially regulated genes in TECs. Differentially regulated genes (≥2 fold) in tracheal epithelial cells after exposure to live Rlow, Rhigh or LAMPs isolated from either strain for 1.5 hours. n = 8 (4 biological replicates x2 dye swap technical replicates) for all microarray experiments. (TIF) [file pone.0112796.s001.tif]

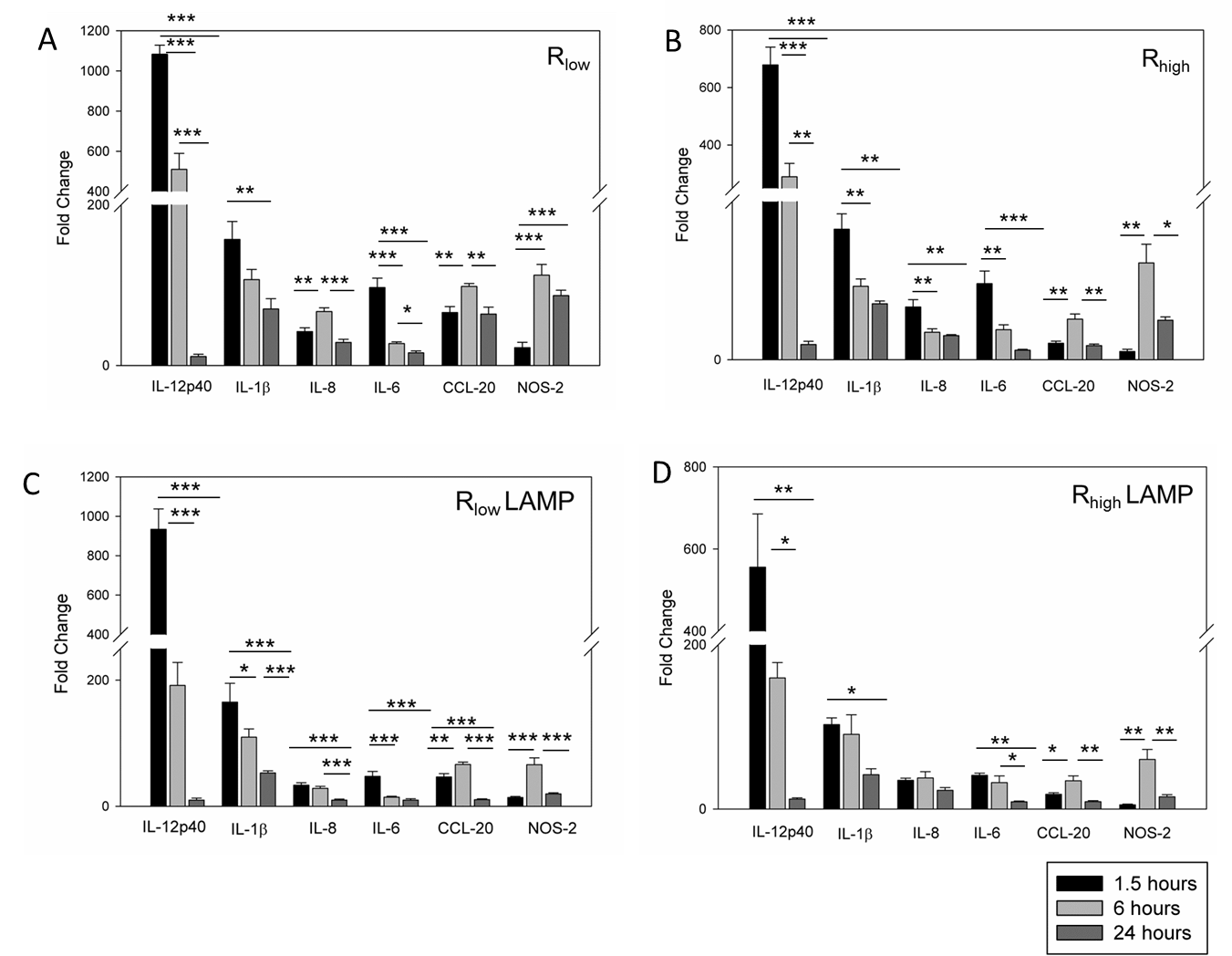

Supplement: Figure S2 — Kinetic analysis of differentially regulated genes encoding inflammatory chemokines and cytokines. Differential gene expression in TECs exposed to Rlow, Rlow LAMP, Rhigh or Rhigh LAMP at 1.5, 6 and 24 hours respectively. Samples normalized to housekeeping gene GAPDH and un-exposed TECs as control. n = 6 for all experiments. Results are denoted as fold change ± SEM with all control values set at 1. Significant differences denoted as * = P<0.05, ** = P<0.01, *** = P<0.001. A: mRNA fold difference in Rlow exposed cells B: mRNA fold difference in Rhigh exposed cells. C: mRNA fold difference in Rlow LAMP exposed cells. D: mRNA fold difference in Rhigh LAMP exposed cells. (TIF) [file pone.0112796.s002.tif]

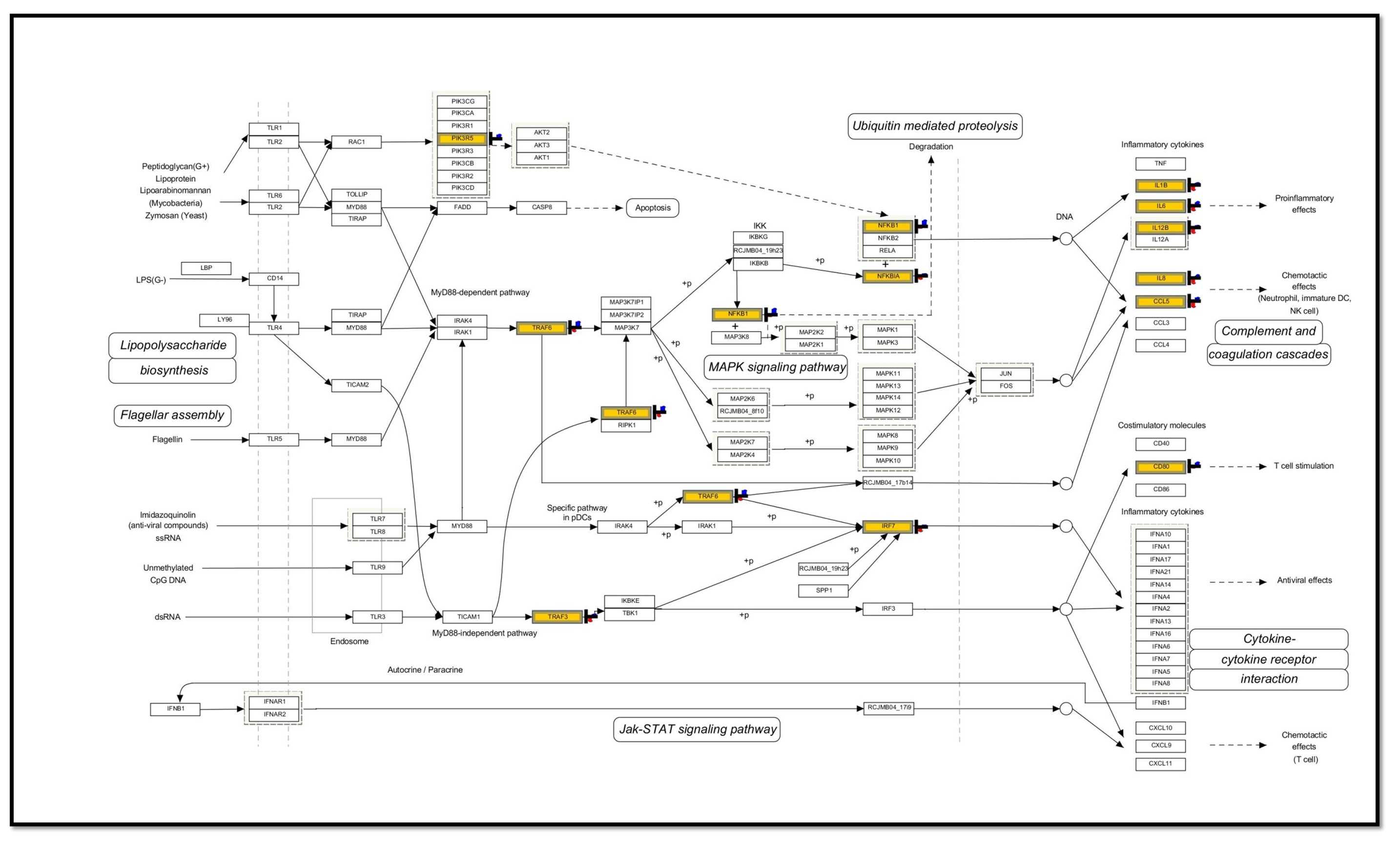

Supplement: Figure S3 — TLR- Signaling pathway. Toll like receptor signaling network: Common genes up-regulated in TECs exposed to Rlow, Rhigh, Rlow LAMP or Rhigh LAMP are depicted in yellow boxes. (TIF) [file pone.0112796.s003.tif]

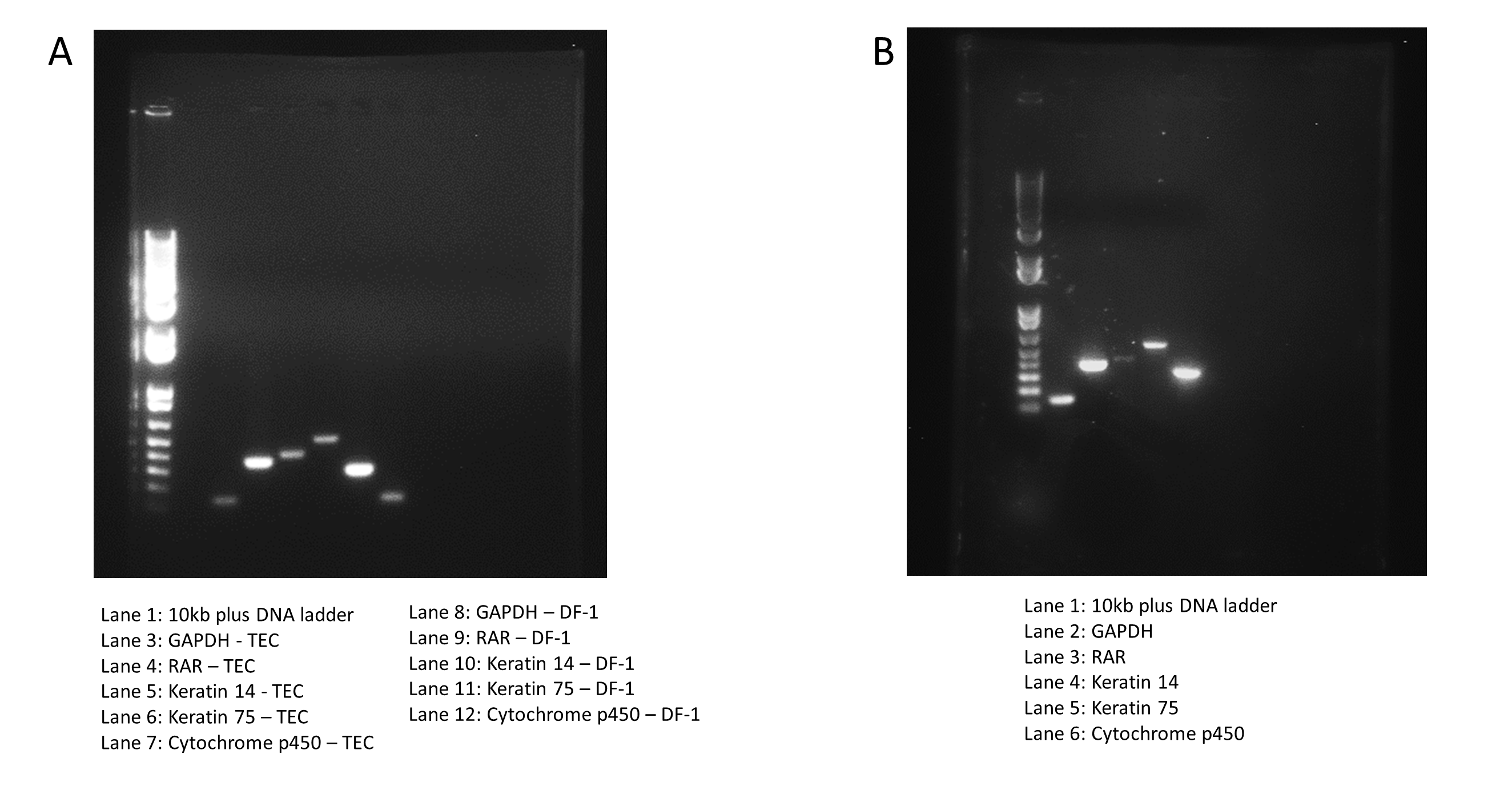

Supplement: Figure S4 — Original agarose gels photos of amplified products of epithelial cell specific genes. 1A: Confirmation of tracheal epithelial cells in culture in vitro using amplification of epithelial cell specific genes compared to chicken embryonic fibroblast (DF-1) cells. 1B: Confirmation of epithelial cell specific genes in freshly isolated epithelial cells from tracheae after ex-vivo exposure. (TIF) [file pone.0112796.s004.tif]
