# Supplementary material for: Mycoplasma gallisepticum Lipid Associated Membrane Proteins Up-regulate Inflammatory Genes in Chicken Tracheal Epithelial Cells via TLR-2 Ligation through an NF-κB Dependent Pathway
Source: PLoS One. 2014 Nov 17;9(11):e112796. doi: 10.1371/journal.pone.0112796 (PMC4234737; doi:10.1371/journal.pone.0112796)
Supplement: Table S1 — Differentially expressed genes (≥2-fold, p-value ≤0.05). Genes commonly up-regulated in TECs exposed to Rlow, Rhigh, Rlow lipoprotein and Rhigh lipoprotein: analyzed for gene ontology hierarchal clustering (Excludes unknown genes listed as finished cDNA clones). (DOCX) [file pone.0112796.s005.docx]

| **Ontological Category** | **Entrez Gene ID** | **Gene Name** | **Fold Change in TECs exposed to** | | | |
| --- | --- | --- | --- | --- | --- | --- |
|  |  |  | **R_low_** | **R_high_** | **R_low_ LAMP** | **R_high_ LAMP** |
| **Immune system processes** | | | | | | |
|  | 768950 | CD80 molecule | 2.96 | 4.77 | 2.44 | 4.02 |
|  | 395082 | Chemokine (C-C motif) ligand 20 | 55.34 | 11.67 | 43.92 | 12.98 |
|  | 417465 | Chemokine (C-C motif) ligand 5 | 2.63 | 2.65 | 2.38 | 2.65 |
|  | 396330 | Interferon regulatory factor 7 | 6.57 | 5.21 | 5.44 | 4.22 |
|  | 395196 | Interleukin 1, beta | 9.89 | 7.86 | 6.75 | 5.65 |
|  | 404671 | Interleukin 12B | 207.80 | 71.80 | 194.67 | 78.21 |
|  | 395337 | Interleukin 6 | 42.33 | 30.57 | 27.31 | 20.95 |
|  | 396495 | Interleukin 8 | 22.92 | 20.72 | 17.87 | 19.44 |
|  | 396093 | Nuclear factor of kappa light polypeptide gene enhancer in B-cells inhibitor, alpha | 5.00 | 4.15 | 5.00 | 3.62 |
|  | 417247 | Similar to TL1A; tumor necrosis factor (ligand) superfamily, member 15 | 4.22 | 14.47 | 4.08 | 10.33 |
|  | 421219 | Toll-like receptor 15 | 2.75 | 2.53 | 3.69 | 2.11 |
|  | 416325 | Granulocyte-macrophage colony-stimulating factor | 5.16 | 8.70 | 5.20 | 9.57 |
| **Regulation of apoptosis** | | | | | | |
|  | 395673 | BCL2-related protein A1 | 4.92 | 4.47 | 4.53 | 3.40 |
|  | 423471 | TNF receptor-associated factor 3 | 3.68 | 5.35 | 4.39 | 5.46 |
|  | 374012 | Baculoviral IAP repeat-containing 2 | 5.42 | 4.07 | 5.45 | 3.48 |
|  | 395196 | Interleukin 1, beta | 9.89 | 7.86 | 6.75 | 5.65 |
|  | 404671 | Interleukin 12B | 207.80 | 71.80 | 194.67 | 78.21 |
|  | 417247 | Similar to TL1A; tumor necrosis factor (ligand) superfamily, member 15 | 4.22 | 14.47 | 4.08 | 10.33 |
|  | 396033 | Nuclear factor of kappa light polypeptide gene enhancer in B-cells 1 | 2.55 | 2.08 | 2.58 | 2.02 |
|  | 396093 | Nuclear factor of kappa light polypeptide gene enhancer in B-cells inhibitor, alpha | 5.00 | 4.15 | 5.00 | 3.62 |
|  | 417319 | Phosphoinositide-3-kinase, regulatory subunit 5, p101 | 6.34 | 3.27 | 5.17 | 3.01 |
| **Signal transduction** | | | | | | |
|  | 419844 | Ras association (ralgds/AF-6) domain family 5 | 5.03 | 3.80 | 4.24 | 3.98 |
|  | 415790 | Ras-related associated with diabetes | 4.88 | 3.03 | 4.27 | 3.25 |
|  | 423471 | TNF receptor-associated factor 3 | 3.68 | 5.35 | 4.39 | 5.46 |
|  | 422884 | TNFAIP3 interacting protein 2 | 10.95 | 4.77 | 7.09 | 7.01 |
|  | 769087 | Angiopoietin-like 4 ANGPTL4 | 7.27 | 2.66 | 5.90 | 2.17 |
|  | 408036 | Epiregulin | 4.01 | 2.02 | 2.80 | 2.66 |
|  | 395196 | Interleukin 1, beta | 9.89 | 7.86 | 6.75 | 5.65 |
|  | 396033 | Nuclear factor of kappa light polypeptide gene enhancer in B-cells 1 | 2.55 | 2.08 | 2.58 | 2.02 |
|  | 396093 | Nuclear factor of kappa light polypeptide gene enhancer in B-cells inhibitor, alpha | 5.00 | 4.15 | 5.00 | 3.62 |
|  | 423290 | Similar to RAS guanyl releasing protein 1 | 2.25 | 3.89 | 3.58 | 3.50 |
|  | 417247 | Similar to TL1A; tumor necrosis factor (ligand) superfamily, member 15 | 4.22 | 14.47 | 4.08 | 10.33 |
|  | 416630 | Suppressor of cytokine signaling 1 | 8.37 | 3.76 | 5.41 | 3.22 |
|  | 421219 | Toll-like receptor 15 | 2.75 | 2.53 | 3.69 | 2.11 |
|  | 378803 | Tumor necrosis factor receptor superfamily, member 11b (osteoprotegerin) | 2.59 | 2.09 | 2.40 | 3.32 |

| **Ontological Category** | **Entrez Gene ID** | **Gene Name** | **Fold Change in TECs exposed to** | | | |
| --- | --- | --- | --- | --- | --- | --- |
|  |  |  | **R_low_** | **R_high_** | **R_low_ LAMP** | **R_high_ LAMP** |
| **Response to stress** | | | | | | |
|  | 395654 | Heparin-binding EGF-like growth factor | 6.66 | 4.39 | 5.22 | 3.61 |
|  | 395196 | Interleukin 1, beta | 9.89 | 7.86 | 6.75 | 5.65 |
|  | 395807 | Nitric oxide synthase 2, inducible | 10.53 | 3.68 | 8.41 | 3.94 |
|  | 418404 | Nuclear factor of kappa light polypeptide gene enhancer in B-cells inhibitor, zeta | 10.24 | 17.96 | 12.14 | 15.32 |
|  | 396424 | Plasminogen activator, urokinase | 7.15 | 16.65 | 7.95 | 14.86 |
|  | 396451 | Prostaglandin-endoperoxide synthase 2 | 12.98 | 10.65 | 13.13 | 9.76 |
|  | 421219 | Toll-like receptor 15 | 2.75 | 2.53 | 3.69 | 2.11 |
|  | 408036 | Epiregulin | 4.01 | 2.02 | 2.80 | 2.66 |
|  | 404671 | Interleukin 12B | 207.80 | 71.80 | 194.67 | 78.21 |
|  | 396093 | Nuclear factor of kappa light polypeptide gene enhancer in B-cells inhibitor, alpha | 5.00 | 4.15 | 5.00 | 3.62 |
| **Cell communication** | | | | | | |
|  | 395820 | Delta-like 1 (Drosophila); similar to C-Delta-1 | 3.19 | 2.76 | 2.18 | 2.37 |
|  | 408036 | Epiregulin | 4.01 | 2.02 | 2.80 | 2.66 |
|  | 395337 | Interleukin 6 | 42.33 | 30.57 | 27.31 | 20.95 |
|  | 420628 | Sorting nexin 10 | 3.71 | 2.02 | 3.31 | 2.25 |
|  | 374168 | Wingless-type MMTV integration site family, member 7A | 3.27 | 4.42 | 2.79 | 4.47 |
| **Cell migration/locomotion** | | | | | | |
|  | 395654 | Heparin-binding EGF-like growth factor | 6.66 | 4.39 | 5.22 | 3.61 |
|  | 395196 | Interleukin 1, beta | 9.89 | 7.86 | 6.75 | 5.65 |
|  | 404671 | Interleukin 12B | 207.80 | 71.80 | 194.67 | 78.21 |
|  | 396424 | Plasminogen activator, urokinase | 7.15 | 16.65 | 7.95 | 14.86 |
|  | 417465 | Chemokine (C-C motif) ligand 5 | 2.63 | 2.65 | 2.38 | 2.65 |
|  | 396495 | Interleukin 8 | 22.92 | 20.72 | 17.87 | 19.44 |
| **Regulation of cell proliferation** | | | | | | |
|  | 768950 | CD80 molecule | 2.96 | 4.77 | 2.44 | 4.02 |
|  | 408036 | Epiregulin | 4.01 | 2.02 | 2.80 | 2.66 |
|  | 395654 | Heparin-binding EGF-like growth factor | 6.66 | 4.39 | 5.22 | 3.61 |
|  | 395196 | Interleukin 1, beta | 9.89 | 7.86 | 6.75 | 5.65 |
|  | 404671 | Interleukin 12B | 207.80 | 71.80 | 194.67 | 78.21 |
|  | 395337 | Interleukin 6 | 42.33 | 30.57 | 27.31 | 20.95 |
|  | 395807 | Nitric oxide synthase 2, inducible | 10.53 | 3.68 | 8.41 | 3.94 |
|  | 396093 | Nuclear factor of kappa light polypeptide gene enhancer in B-cells inhibitor, alpha | 5.00 | 4.15 | 5.00 | 3.62 |
|  | 396424 | Plasminogen activator, urokinase | 7.15 | 16.65 | 7.95 | 14.86 |
|  | 396451 | Prostaglandin-endoperoxide synthase 2 | 12.98 | 10.65 | 13.13 | 9.76 |
|  | 417247 | Similar to TL1A; tumor necrosis factor (ligand) superfamily, member 15 | 4.22 | 14.47 | 4.08 | 10.33 |

| **Ontological Category** | **Entrez Gene ID** | **Gene Name** | **Fold change in TECs exposed to** | | | |
| --- | --- | --- | --- | --- | --- | --- |
|  |  |  | **R_low_** | **R_high_** | **R_low_ LAMP** | **R_high_ LAMP** |
| **Response to stimulus** | | | | | | |
|  | 395082 | Chemokine (C-C motif) ligand 20 | 55.34 | 11.67 | 43.92 | 12.98 |
|  | 417465 | Chemokine (C-C motif) ligand 5 | 2.63 | 2.65 | 2.38 | 2.65 |
|  | 395654 | Heparin-binding EGF-like growth factor | 6.66 | 4.39 | 5.22 | 3.61 |
|  | 418812 | Immunoresponsive 1 homolog (mouse) IRG1 | 47.48 | 28.24 | 43.40 | 29.42 |
|  | 396330 | Interferon regulatory factor 7 | 6.57 | 5.21 | 5.44 | 4.22 |
|  | 395196 | Interleukin 1, beta | 9.89 | 7.86 | 6.75 | 5.65 |
|  | 404671 | Interleukin 12B | 207.80 | 71.80 | 194.67 | 78.21 |
|  | 395337 | Interleukin 6 | 42.33 | 30.57 | 27.31 | 20.95 |
|  | 396495 | Interleukin 8 | 22.92 | 20.72 | 17.87 | 19.44 |
|  | 395807 | Nitric oxide synthase 2, inducible | 10.53 | 3.68 | 8.41 | 3.94 |
|  | 396093 | Nuclear factor of kappa light polypeptide gene enhancer in B-cells inhibitor, alpha | 5.00 | 4.15 | 5.00 | 3.62 |
|  | 418404 | Nuclear factor of kappa light polypeptide gene enhancer in B-cells inhibitor, zeta | 10.24 | 17.96 | 12.14 | 15.32 |
|  | 396424 | Plasminogen activator, urokinase | 7.15 | 16.65 | 7.95 | 14.86 |
|  | 396451 | Prostaglandin-endoperoxide synthase 2 | 12.98 | 10.65 | 13.13 | 9.76 |
|  | 417247 | Similar to TL1A; tumor necrosis factor (ligand) superfamily, member 15 | 4.22 | 14.47 | 4.08 | 10.33 |
|  | 421219 | Toll-like receptor 15 | 2.75 | 2.53 | 3.69 | 2.11 |
| **Receptor binding** | | | | | | |
|  | 769087 | Angiopoietin-like 4 ANGPTL4 | 7.27 | 2.66 | 5.90 | 2.17 |
|  | 395082 | Chemokine (C-C motif) ligand 20 | 55.34 | 11.67 | 43.92 | 12.98 |
|  | 417465 | Chemokine (C-C motif) ligand 5 | 2.63 | 2.65 | 2.38 | 2.65 |
|  | 395820 | Delta-like 1 (Drosophila); similar to C-Delta-1 | 3.19 | 2.76 | 2.18 | 2.37 |
|  | 408036 | Epiregulin | 4.01 | 2.02 | 2.80 | 2.66 |
|  | 395654 | Heparin-binding EGF-like growth factor | 6.66 | 4.39 | 5.22 | 3.61 |
|  | 395196 | Interleukin 1, beta | 9.89 | 7.86 | 6.75 | 5.65 |
|  | 404671 | Interleukin 12B | 207.80 | 71.80 | 194.67 | 78.21 |
|  | 395337 | Interleukin 6 (interferon, beta 2) | 42.33 | 30.57 | 27.31 | 20.95 |
|  | 396495 | Interleukin 8 | 22.92 | 20.72 | 17.87 | 19.44 |
|  | 417247 | Similar to TL1A; tumor necrosis factor (ligand) superfamily, member 15 | 4.22 | 14.47 | 4.08 | 10.33 |
| **Protein binding** | | | | | | |
|  | 395673 | BCL2-related protein A1 | 4.92 | 4.47 | 4.53 | 3.40 |
|  | 768950 | CD80 molecule | 2.96 | 4.77 | 2.44 | 4.02 |
|  | 420343 | F-box protein 32 | -2.95 | -3.37 | -3.59 | -3.77 |
|  | 419844 | Ras association (ralgds/AF-6) domain family 5 | 5.03 | 3.80 | 4.24 | 3.98 |
|  | 423471 | TNF receptor-associated factor 3 | 3.68 | 5.35 | 4.39 | 5.46 |
|  | 422884 | TNFAIP3 interacting protein 2 | 10.95 | 4.77 | 7.09 | 7.01 |
|  | 769087 | Angiopoietin-like 4 ANGPTL4 | 7.27 | 2.66 | 5.90 | 2.17 |
|  | 374012 | Baculoviral IAP repeat-containing 2 | 5.42 | 4.07 | 5.45 | 3.48 |
|  | 395082 | Chemokine (C-C motif) ligand 20 | 55.34 | 11.67 | 43.92 | 12.98 |
|  | 417465 | Chemokine (C-C motif) ligand 5 | 2.63 | 2.65 | 2.38 | 2.65 |
|  | 395820 | Delta-like 1 (Drosophila); similar to C-Delta-1 | 3.19 | 2.76 | 2.18 | 2.37 |
|  | 417515 | Deltex homolog 2 (Drosophila) | 3.72 | 2.34 | 2.99 | 2.57 |
|  | 408036 | Epiregulin | 4.01 | 2.02 | 2.80 | 2.66 |
|  | 421747 | Fyn-related kinase | 3.17 | 3.69 | 2.62 | 3.57 |
|  | 395654 | Heparin-binding EGF-like growth factor | 6.66 | 4.39 | 5.22 | 3.61 |
|  | 396330 | Interferon regulatory factor 7 | 6.57 | 5.21 | 5.44 | 4.22 |

| **Ontological Category** | **Entrez Gene ID** | **Gene Name** | | **Fold change in TECs exposed to** | | | |
| --- | --- | --- | --- | --- | --- | --- | --- |
|  |  |  |  | **R_low_** | **R_high_** | **R_low_ LAMP** | **R_high_ LAMP** |
| **Protein binding continued.** | | | | | | | |
|  | 395196 | Interleukin 1, beta | | 9.89 | 7.86 | 6.75 | 5.65 |
|  | 404671 | Interleukin 12B | | 207.80 | 71.80 | 194.67 | 78.21 |
|  | 422219 | Interleukin 13 receptor, alpha 2 | | 5.40 | 3.25 | 4.57 | 3.64 |
|  | 421686 | Interleukin 20 receptor, alpha | | 5.01 | 3.73 | 3.72 | 3.97 |
|  | 424704 | Interleukin 23 receptor | | 2.34 | 2.41 | 2.48 | 4.44 |
|  | 395337 | Interleukin 6 (interferon, beta 2) | | 42.33 | 30.57 | 27.31 | 20.95 |
|  | 396495 | Interleukin 8 | | 22.92 | 20.72 | 17.87 | 19.44 |
|  | 395807 | Nitric oxide synthase 2, inducible | | 10.53 | 3.68 | 8.41 | 3.94 |
|  | 396033 | Nuclear factor of kappa light polypeptide gene enhancer in B-cells 1 | | 2.55 | 2.08 | 2.58 | 2.02 |
|  | 396093 | Nuclear factor of kappa light polypeptide gene enhancer in B-cells inhibitor, alpha | | 5.00 | 4.15 | 5.00 | 3.62 |
|  | 424586 | Polo-like kinase 3 | | 4.70 | 2.83 | 2.45 | 2.04 |
|  | 417247 | Similar to TL1A; tumor necrosis factor (ligand) superfamily, member 15 | | 4.22 | 14.47 | 4.08 | 10.33 |
|  | 423163 | Similar to TRAF6; TNF receptor-associated factor 6 | | 3.35 | 3.01 | 3.29 | 2.24 |
|  | 420628 | Sorting nexin 10 | | 3.71 | 2.02 | 3.31 | 2.25 |
|  | 416630 | Suppressor of cytokine signaling 1 | | 8.37 | 3.76 | 5.41 | 3.22 |
|  | 421219 | Toll-like receptor 15 | | 2.75 | 2.53 | 3.69 | 2.11 |
|  | 419088 | Tsukushin | | 4.24 | 2.68 | 3.76 | 3.02 |
|  | 378803 | Tumor necrosis factor receptor superfamily, member 11b (osteoprotegerin) | | 2.59 | 2.09 | 2.40 | 3.32 |
|  | 396250 | V-ets erythroblastosis virus E26 oncogene homolog 2 (avian) | | 2.53 | 2.75 | 2.13 | 2.43 |
|  | 374168 | Wingless-type MMTV integration site family, member 7A | | 3.27 | 4.42 | 2.79 | 4.47 |
| **Other genes** | | | | | | | |
|  | 420838 | CD83 molecule | | 5.98 | 4.53 | 5.63 | 3.75 |
|  | 428492 | F-box and leucine-rich repeat protein 7 | | 3.30 | 4.23 | 3.29 | 4.07 |
|  | 420343 | F-box protein 32 | | -2.95 | -3.37 | -3.59 | -3.77 |
|  | 422061 | G protein-coupled receptor 116 | | 3.57 | 3.99 | 3.68 | 3.21 |
|  | 416546 | NADPH oxidase organizer 1 | | 31.41 | 11.36 | 27.79 | 11.96 |
|  | 395138 | ST3 beta-galactoside alpha-2,3-sialyltransferase 6 | | 3.33 | 4.85 | 3.07 | 4.08 |
|  | 771693 | TNFAIP3 interacting protein 3 | | 5.04 | 2.69 | 5.87 | 2.81 |
|  | 427985 | UDP-Gal:betaglcnac beta 1,3-galactosyltransferase, polypeptide 5 | | 2.21 | 3.42 | 2.56 | 3.78 |
|  | 418422 | Chromosome 3 open reading frame 52 | | 2.45 | 2.39 | 2.22 | 2.10 |
|  | 417515 | Deltex homolog 2 | | 3.72 | 2.34 | 2.99 | 2.57 |
|  | 769474 | Family with sequence similarity 83, member C | | 2.77 | 3.92 | 2.42 | 3.78 |
|  | 421747 | Fyn-related kinase | | 3.17 | 3.69 | 2.62 | 3.57 |
|  | 417968 | Guanylate cyclase 2C | | 2.10 | 2.46 | 2.01 | 2.16 |
|  | 422840 | Heparan sulfate (glucosamine) 3-O-sulfotransferase 1 | | 3.50 | 2.94 | 3.22 | 2.72 |
|  | 423723 | Peptidylprolyl isomerase F | | 4.28 | 2.33 | 2.44 | 2.11 |
|  | 423227 | Phospholipase A2, group IVE | | 2.45 | 2.65 | 3.18 | 2.44 |
|  | 424586 | Polo-like kinase 3 (Drosophila) | | 4.70 | 2.83 | 2.45 | 2.04 |
|  | 428650 | Radical S-adenosyl methionine domain containing 2 | | 2.85 | 2.43 | 2.30 | 3.36 |
|  | 419262 | Regulator of telomere elongation helicase 1 | | 2.78 | 3.65 | 2.27 | 2.95 |
| **Ontological Category** | **Entrez Gene ID** | **Gene Name** | | **Fold change in tecs exposed to** | | | |
|  |  |  |  | **R_low_** | **R_high_** | **R_low_ LAMP** | **R_high_ LAMP** |
| **Other genes continued.** | | | | | | | |
|  | 431250 | Similar to EBI 2: EBV induced G-protein coupled receptor | 4.10 | | 3.84 | 2.45 | 2.27 |
|  | 422654 | Similar to K60 protein | 27.79 | | 24.75 | 12.97 | 20.83 |
|  | 770634 | Similar to fibrinogen silencer binding protein | 3.93 | | 5.48 | 2.66 | 4.14 |
|  | 420628 | Sorting nexin 10 | 3.71 | | 2.02 | 3.31 | 2.25 |
|  | 423477 | Tumor necrosis factor, alpha-induced protein 2 | 9.29 | | 4.90 | 8.42 | 4.39 |
|  | 421684 | Tumor necrosis factor, alpha-induced protein 3 | 4.98 | | 4.25 | 4.28 | 3.21 |
|  | 420942 | Uridine phosphorylase 1 | 2.64 | | 2.37 | 2.27 | 2.90 |
|  | 419618 | Zinc finger CCCH-type containing 12A | 4.16 | | 3.33 | 4.22 | 3.16 |
|  | 396384 | Interferon regulatory factor 1 (IRF1) | 5.66 | | 5.48 | 4.73 | 5.07 |
|  |  |  |  | |  |  |  |
|  | 395827 | Cytochrome P450, family 24, subfamily A, polypeptide 1 (CYP24A1) | 3.06 | | 3.17 | 3.30 | 3.17 |
