# Supplementary material for: Mycoplasma gallisepticum Lipid Associated Membrane Proteins Up-regulate Inflammatory Genes in Chicken Tracheal Epithelial Cells via TLR-2 Ligation through an NF-κB Dependent Pathway
Source: PLoS One. 2014 Nov 17;9(11):e112796. doi: 10.1371/journal.pone.0112796 (PMC4234737; doi:10.1371/journal.pone.0112796)
Supplement: Table S2 — Differentially expressed unique genes in Rlow exposed TECs (≥5-fold, p-value ≤0.05) (Excludes unknown genes listed as finished cDNA clones). (DOCX) [file pone.0112796.s006.docx]

| **Entrez Gene ID** | **Gene Name** | **Fold change in R_low_ exposed TECs** |
| --- | --- | --- |
|  |  |  |
| 395888 | NK2 transcription factor related, locus 3 | 87.83 |
| 428065 | Protocadherin 8 | 65.12 |
|  | Partial mRNA for immunoglobulin heavy chain variable region, clone U11B3. [AM773275] | 33.71 |
| 418236 | Parvin gamma | 29.20 |
| 429104 | Cyclin-dependent kinase inhibitor 2C | 28.81 |
| 427105 | POLR3G polymerase (RNA) III (DNA directed) polypeptide G (32kD) | 19.60 |
| 374049 | Tumor protein D52-like 1 | 19.52 |
| 378791 | Reticulon 1 | 19.15 |
| 414343 | Gal 9 | 15.95 |
| 374004 | Myogenin (myogenic factor 4) | 13.33 |
| 416953 | Member RAS oncogene family similar to small GTP-binding protein Rab36 | 12.48 |
| 424940 | Cholinergic receptor, nicotinic, delta | 11.74 |
| 424171 | G6PC2 glucose-6-phosphatase, catalytic, 2 | 9.32 |
| 428714 | Probable tRNA threonylcarbamoyladenosine biosynthesis protein YwlC-like | 7.55 |
| 408183 | Cytochrome P450, family 26, subfamily A, polypeptide 1 | 7.25 |
| 427503 | FSD2 fibronectin type III and SPRY domain containing 2 | 7.10 |
| 428247 | HTR3A 5-hydroxytryptamine (serotonin) receptor 3A, ionotropic | 7.04 |
| 373928 | Peroxisome proliferator-activated receptor gamma | 6.88 |
| 429283 | Distal-less homeobox 1 | 6.36 |
| 426186 | Transmembrane protein 106C | 6.26 |
| 768668 | Arylacetamide deacetylase-like 4-like | 6.13 |
| 396260 | Mature avidin | 6.07 |
| 396114 | Pyrimidinergic receptor P2Y, G-protein coupled, 6 | 5.95 |
| 396300 | Kainate binding protein | 5.87 |
| 427718 | Leukemia inhibitory factor | 5.78 |
| 395725 | Deoxyribonuclease I | 5.77 |
| 422307 | Immune-responsive gene 1 protein-like | 5.69 |
| 415820 | Hepatic nuclear factor 4beta | 5.63 |
| 416996 | Inositol polyphosphate-5-phosphatase J | 5.61 |
| 422219 | Interleukin 13 receptor, alpha 2 | 5.39 |
| 417505 | Hydroxy-delta-5-steroid dehydrogenase, 3 beta- and steroid delta-isomerase 7 | 5.34 |
| 768877 | SAG S-antigen; retina and pineal gland (arrestin) | 5.19 |
| 428191 | Ciliary rootlet coiled-coil, rootletin | 5.18 |
| 427028 | Olfactory receptor 14J1 like | 5.13 |
| 429337 | Apelin receptor | 5.10 |
| 419844 | Ras association (RalGDS/AF-6) domain family 5 | 5.03 |
| 430670 | Chymotrypsin C | 5.03 |
| 419751 | Glutamate receptor, ionotropic, kainate 4 | 5.01 |
| 421686 | Interleukin 20 receptor, alpha | 5.01 |
